# Description of probiotic use in preterm infants in England and Wales 2016–2022

**DOI:** 10.1136/bmjpo-2025-003605

**Published:** 2025-07-24

**Authors:** Alice Aveline, Lisa Szatkowski, Janet Berrington, Kate Costeloe, Alex Bottle, Shalini Ojha, Paul Fleming, Cheryl Battersby

**Affiliations:** 1 Neonatal Medicine, School of Public Health, Faculty of Medicine, Imperial College London, London, UK; 2 Centre for Paediatrics and Child Health, Imperial College London, London, UK; 3 Centre for Perinatal Research, Lifespan and Population Health, University of Nottingham School of Medicine, Nottingham, UK; 4 Newcastle Upon Tyne Hospitals NHS Foundation Trust, Newcastle Upon Tyne, UK; 5 Translational and Clinical Research Institute, Newcastle University, Newcastle upon Tyne, UK; 6 Queen Mary University of London, London, UK; 7 Department of Neonatology, Homerton University Hospital NHS Foundation Trust, London, UK; 8 Department of Primary Care and Public Health, Imperial College London, London, UK; 9 Centre for Perinatal Research, Lifespan and Population Health, School of Medicine, University of Nottingham, Derby, UK; 10 Neonatal Unit, University Hospitals of Derby and Burton NHS Foundation Trust, Derby, UK

**Keywords:** Epidemiology, Infant, Neonatology

## Abstract

**Objective:**

To describe the use of probiotics among preterm infants in neonatal units and explore factors that influence exposure.

**Design:**

Observational study using prospectively recorded health data.

**Setting:**

England and Wales.

**Patients:**

48 048 infants born at <32 weeks gestational age (GA) and admitted to a neonatal unit between 1 January 2016 and 31 December 2022.

**Main outcome measures:**

Measures of probiotic use (number and proportion of infants exposed to probiotics, postnatal age of first probiotic exposure and discontinuation).

**Results:**

The proportion of infants who received probiotics increased from 9% to 54% over the study period. Median GA of infants given probiotics was 29^+3^ weeks (IQR 27^+3^–30^+6^). Probiotics were started on median day 5 (IQR 2–8), earlier for those born at >28 weeks GA (median day 4, IQR 2–7), most frequently after enteral feeds (66% of exposed infants) and were usually discontinued between 32 and 36 weeks postmenstrual age (PMA) (47% at 32^+0^–33^+6^ weeks PMA, 33% at 34^+0^–35^+6^ weeks PMA). Among infants cared for in probiotic neonatal intensive care units (defined as units where 50% or more infants born <32 weeks gestation were exposed to probiotics), 23% were never given probiotics. Infants from whom probiotics were withheld had a lower gestational age, lower birth weight z score and higher illness severity score or were more mature.

**Conclusions:**

By 2022, over half of infants born at <32 weeks GA were exposed to probiotics, but almost one quarter did not receive them despite being in a probiotic unit. Our findings help inform the interpretation of observational data and the design of future studies addressing the continued uncertainty around the safety and efficacy of probiotics.

**WHAT IS ALREADY KNOWN ON THIS TOPIC**

- Survey data shows that UK neonatal units are increasingly adopting guidelines which recommend routine use of probiotics in infants born before 32 weeks gestation.

**WHAT THIS STUDY ADDS**

- This study, using baby and unit-level population data, found that, in line with survey data, just over half of infants, born before 32 weeks gestation in 2022, received probiotics in the first 14 days of life. However, what was previously unknown is that, in tertiary neonatal intensive care units that routinely use probiotics, almost a quarter of infants do not receive probiotics; these infants tend to be the smallest and the sickest.

**HOW THIS STUDY MIGHT AFFECT RESEARCH, PRACTICE OR POLICY**

- The study findings reflect some equipoise among UK clinicians and support the need for additional research examining the efficacy and safety of probiotics in the preterm population. Findings from this study will help inform the design of future studies.

## Introduction

 There has been sustained interest in evaluating whether probiotics can diminish or prevent major complications associated with preterm birth.^1^ Studies have focused on the potential for probiotics to reduce the incidence of necrotising enterocolitis (NEC) and late onset sepsis (LOS). These conditions are recognised as among the leading causes of death in preterm infants, especially in those born at <32 weeks gestational age (GA).^2^ The most recent Cochrane meta-analysis^3^ showed that probiotics may reduce the risk of NEC (RR 0.54, 95% CI 0.46 to 0.65; 57 trials, 10 918 infants; low certainty); probably reduce mortality (RR 0.77, 95% CI 0.66 to 0.90; 54 trials, 10 484 infants; moderate certainty); and have little or no effect on the risk of late‐onset invasive infection (RR 0.89, 95% CI 0.82 to 0.97; 49 trials, 9876 infants; moderate certainty).

Despite evidence from the 57 trials evaluating NEC that were included in the Cochrane review, recommendations vary. In 2020, the European Society for Paediatric Gastroenterology, Hepatology and Nutrition (ESPGHAN) made conditional recommendations that certain probiotic strains and probiotic combinations could be used as potential preventative strategies for NEC.^4^ The American Academy of Pediatrics issued a statement in 2021 which did not support routine universal probiotic administration, especially for infants <1000 g, citing a lack of evidence of benefit in trials and limited availability of suitable pharmaceutical grade products.^5^ More recently, the US Federal Drug Administration (FDA) has warned manufacturers about the marketing of probiotic products to treat or prevent diseases in preterm infants in the absence of regulatory approval for this use.^6^ This has led to a large number of US neonatal units halting probiotic use.^7^ Inconsistent evidence from clinical trials, combined with conflicting guidance from professional bodies, has contributed to considerable clinician uncertainty about whether or not probiotics should be used in routine neonatal practice.^8^

In the UK, neonatal services are organised in geographically defined operational delivery networks (ODNs) and infants are transferred between neonatal units, within an ODN, to receive appropriate care. Neonatal units are divided into three types: neonatal intensive care units (NICUs) (level 3), local neonatal units (level 2) and special care units (level 1). All infants born before 27 weeks gestation and multiple pregnancies delivered before 28 weeks gestation receive their early days care in a NICU. Probiotic guidelines vary within and between networks. In 2022, Patel *et al*^9^ conducted a survey of probiotic use in UK neonatal units which reported that 44% units (n=70/161) routinely used probiotics in the population of preterm infants born at <32 weeks GA. The same study found that the two most commonly used probiotics in the UK are a combination of *Bifidobacterium infantis, Streptococcus thermophilus* and *Bifidobacterium lactis* (abbreviated as PP) and a product containing *Lactobacillus acidophilus*, *Bifidobacterium bifidum* and *Bifidobacterium longum* spp *infantis* (abbreviated as LB). While survey results can be helpful to explore differences in practice, they may not reflect adherence by individual caregivers to unit guidelines at an infant level. Furthermore, as infants are commonly transferred between units, surveys do not necessarily reflect an infant’s whole journey through neonatal care. In this study, we aimed to describe patterns of probiotic use at unit and infant level and explore factors that may influence probiotic exposure within units that routinely use probiotics.

## Methods

### Study design

Retrospective cohort study using routinely collected data held in the National Neonatal Research Database (NNRD).

### Data source

The NNRD contains records for all infants admitted to NHS neonatal units in England and Wales. Clinicians enter data daily into their electronic patient record system as part of routine care. NNRD records include demographic details (e.g. maternal conditions, gestation, birth weight), daily information (e.g. medications and respiratory support), test results (e.g. ultrasound scan findings, abdominal X-ray findings) and diagnoses.^10^

### Eligibility criteria

#### Inclusion criteria

Infants born at less than 32 weeks GA between 1 January 2016 and 31 December 2022 (7 years) and admitted to a neonatal unit in England and Wales.

#### Exclusion criteria

Infants with data missing for any of GA, birth weight or birth year; first admission data missing or beginning after three postnatal days; birth weight for GA z score exceeds an absolute value of 4; died within the first 4 days of life; or born with a major congenital abnormality (online supplemental material 1).

### Definitions

#### Exposure

Probiotic exposure: Infants were classed as probiotic recipients if there was documented receipt in the NNRD ‘daily drugs’ field of any probiotic for at least 1 day. Documented probiotics included: Labinic (also known as LB2 and hereafter abbreviated to as LB), Proprems (PP), Infloran, Bio-kult and an unspecified Bifidobacterium (online supplemental material 2 for product details). Infants whose NNRD records showed only 1 day of probiotic exposure were classed as recipients if they were cared for in a unit that routinely used probiotics (see below).

We recorded first exposure by postnatal day and reported results in two categories: those who first received probiotics on or before postnatal day 14 and those who first received probiotics after day 14. We chose a 14 day cut-off^11^ because we wished to capture the time at which most infants will have been enterally fed and receipt of probiotics may mechanistically impact NEC.^12 13^

### Analysis exploring factors influencing probiotic exposure in a ‘probiotic unit’

The main determinant of an infant’s probiotic exposure status is whether the unit providing care had implemented a guideline for the routine use of probiotics among preterm infants. To explore other factors that may influence an infant’s exposure to probiotics, we conducted a second analysis excluding units that do not use probiotics routinely. We limited this analysis to ‘Probiotic NICUs’, defined as NICUs where either the existence of a probiotic guideline was confirmed by that NICU in a survey^11^ or data analysis confirmed that more than half the infants <32 weeks admitted in the same month received probiotics.^14^ As NICUs care for larger numbers of very preterm infants each month than local neonatal units or special care units, we could be more confident of correctly classifying the probiotic status of NICUs than for units with a smaller monthly denominator. We contacted units where there was uncertainty about their probiotic status from the NNRD data, to verify their probiotic status (online supplemental material 3). Infants cared for in a probiotic NICU on postnatal day 3 were included in this analysis. We used the infant’s location on postnatal day 3 as most transfers to a higher level unit occur before this.

#### Other factors

The other variables explored were infant background variables (sex, gestational age, birth weight z score, birth year, multiple birth, mode of delivery, intra-uterine growth restriction (defined as birth weight for age z score <-2 SD)), maternal variables (ethnicity, gravidity, maternal infection, chorioamnionitis, antenatal steroids, Index of Multiple Deprivation quintile (a broad measure of social deprivation^15^)) and variables relating to the infant’s postnatal condition and care (surfactant receipt in first 24 hours, illness severity score in first 2 days (online supplemental material 4), receipt of enteral milk in the first 4 days, highest level of care in the first 4 days (using the BAPM categorisation)^16^ and transfer between units (within 72 hours)). Details of the definitions and coding for these variables are shown in online supplemental material 5.

### Statistical methods

We report descriptive statistics for infant and maternal background characteristics and details of early clinical care by probiotic exposure (any vs no exposure). For all probiotic-exposed infants, we describe the same characteristics categorised by the timing of first probiotic exposure (first exposed before or at postnatal day 14 vs first exposed after day 14). For infants cared for in NICUs that used probiotics, we describe the characteristics of the infants by probiotic exposure status (never exposed, first exposed within the first 14 days and first exposed after day 14). We also describe the strains of probiotics used over time, and timing of commencement and discontinuation. We tested the impact of GA (< 28 weeks vs >=28 weeks) on the timing of commencement of probiotics using a χ2 test.

For infants cared for in a probiotic NICU, we examined variables that were associated with probiotic status using unadjusted, multinomial logistic regression. Due to the large cohort size, even small differences in the distribution of background variables are likely to be statistically significant. We therefore used effect size measures (Cohen’s d and Cohen’s ω) to determine which variables should be included in the final multivariable model. Independent variables were included in the full multivariable model if, in the unadjusted analysis, they had a substantial effect on probiotic status (defined as any Cohen’s d exceeded 0.2 for continuous variables or Cohen’s ω exceeded 0.1 for categorical variables).^17^ The identity of the unit that cared for the infant on postnatal day 3 was also included in the full model as an independent variable, to control for differences between units. We conducted a series of likelihood ratio tests comparing a reduced and the full multivariable, multinomial, logistic regression model. The reduced model contained the same independent variables as the full model with the deletion of the background variable of interest.

### Ethics

Ethics approval was granted by the South-East Scotland Research Ethics Committee 01 (REC reference 23/SS/0016) for use of the NNRD data as part of a larger study evaluating the impact of introduction of a care bundle on incidence of NEC.^18^

### Patient and public involvement

Prior to data extraction, focus group sessions with parents, former NICU patients and advocacy groups were held to establish perspectives on the use of routinely collected neonatal data to monitor probiotic efficacy. The focus group was not involved in the design of this study. In addition, the NNRD board includes two members who are parents of former NICU patients.

## Results

### Numbers and proportions of infants receiving probiotics over time and by type

**Figure 1:**
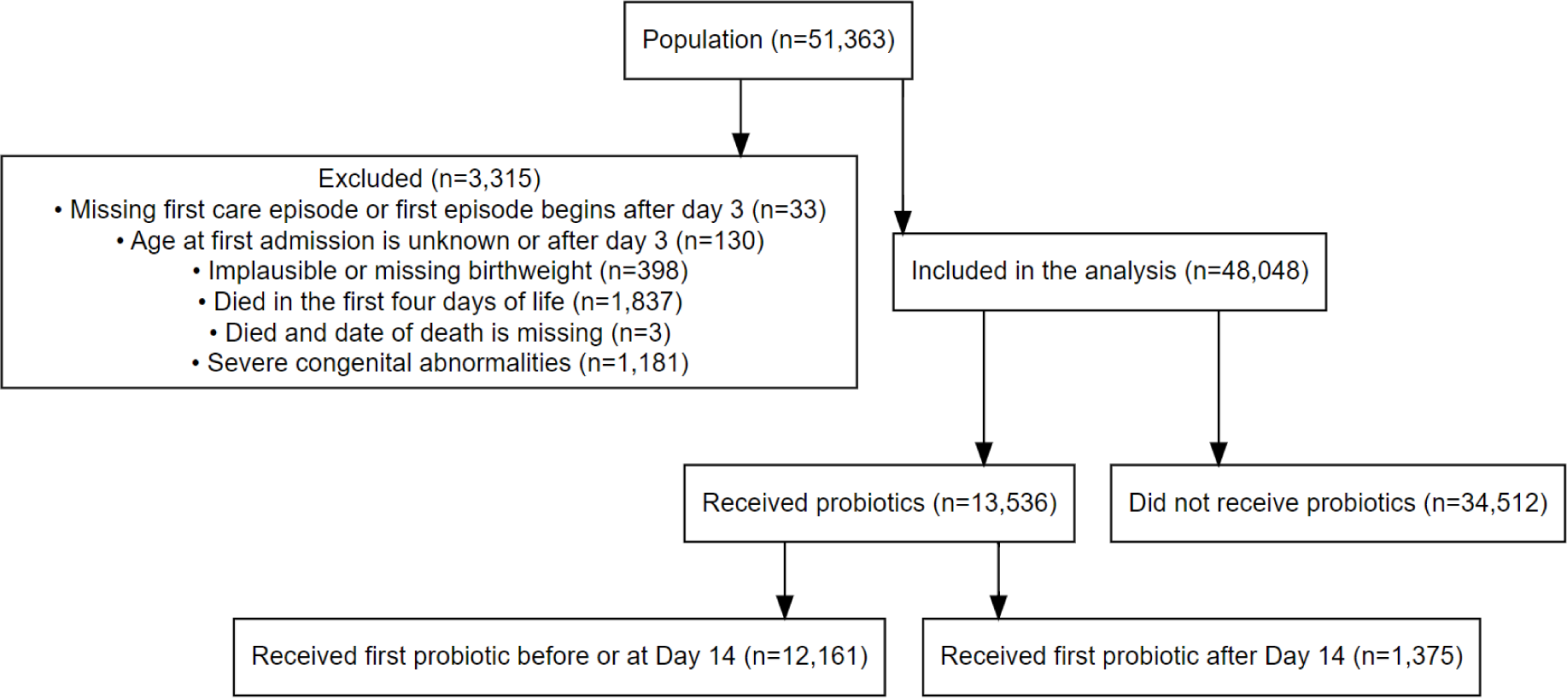
Application of exclusion criteria.

**Figure 2:**
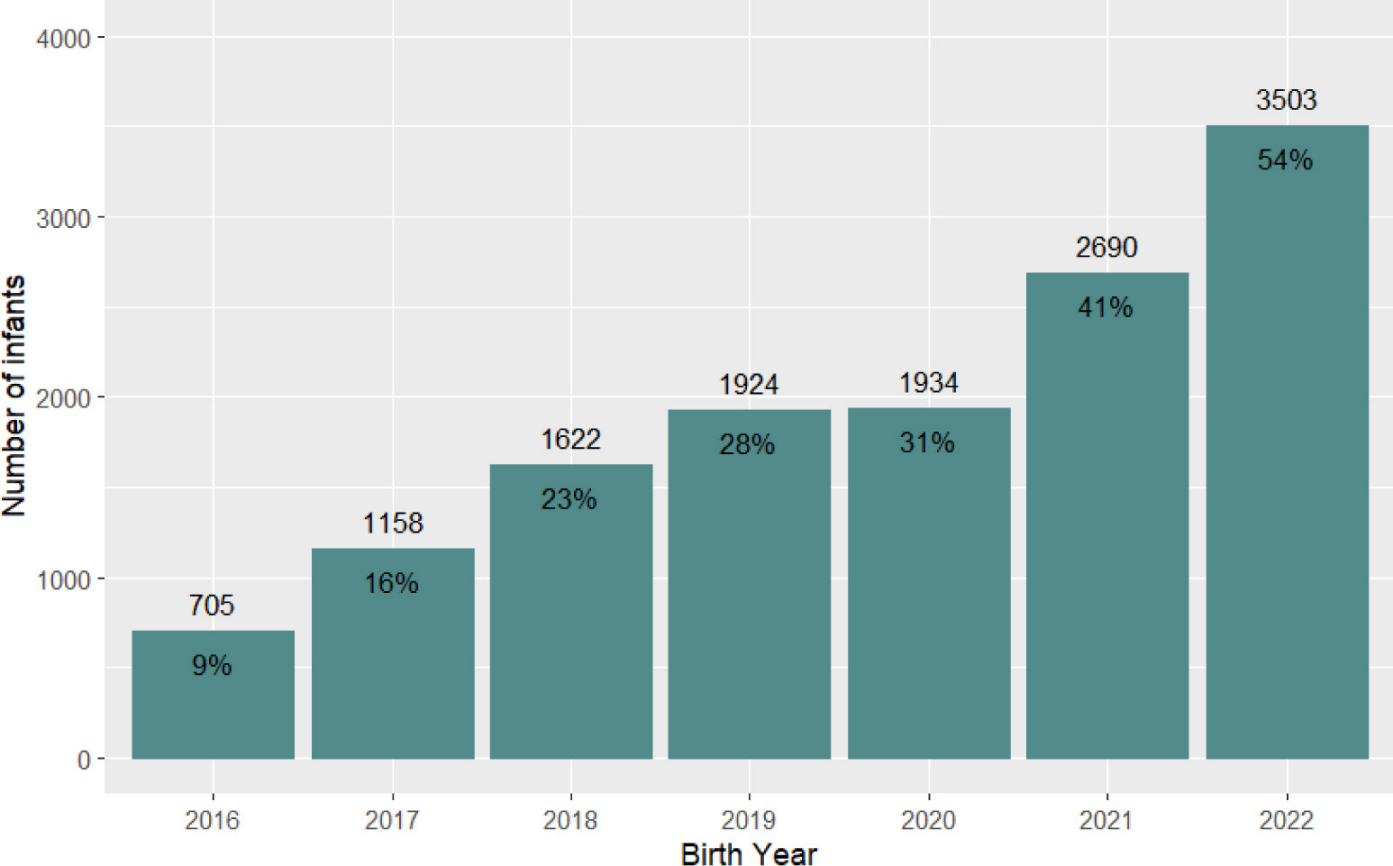
Number and proportion of infants who received probiotics by birth year.

Table 1 reports the clinical characteristics in the whole cohort stratified by probiotic receipt. Following exclusions (figure 1), 48 048 of 51 363 infants born <32 weeks GA were included in the analyses of whom 13 536 (28.2%) received probiotics. Of these 12 161 (89.8%) commenced probiotics within the first 14 postnatal days. Online supplemental table 1 shows the background characteristics of the infants who received probiotics stratified by the time when probiotics were first received (on or before day 14 vs after day 14). The proportion of infants classified as probiotic recipients increased from 9.4% to 54.4% between 2016 and 2022 (figure 2).

**Table 1:** Background characteristics and early clinical care of the cohort stratified by probiotic status

	Whole cohort (n=48 048)		Probiotic NICUs (n=11 262)		
	Never exposed to probiotic	Probiotic exposed	Never exposed to probiotic	First exposed ≤ day 14	First exposed > day 14
Number of infants (%)	34 512 (71.8%)	13 536 (28.2%)	2561 (22.7%)	8081 (71.8%)	620 (5.5%)
**Infant background variables**					
Male sex n (%)	18 941 (54.9)	7406 (54.7)	1438 (56.2)	4396 (54.4)	326 (52.6)
Gestational age at birth median (IQR)	29.7 (27.6–31.0)	29.0 (27.0–30.6)	28.9 (26.4–30.7)	28.6 (26.6–30.4)	26.6 (25.1–28.4)
Birth weight (grams) median (IQR)	1240 (935-1,520)	1140 (864-1,440)	1130 (810-1,460)	1090 (825-1,390)	835 (680–1060)
Birth year n (%)					
2016–2019	23 371 (67.7)	5409 (40.0)	1767 (69.0)	3347 (41.4)	256 (41.3)
2020–2022	11 141 (32.3)	8127 (60.0)	794 (31.0)	4734 (58.6)	364 (58.7)
Multiple birth n (%)	8480 (24.6)	3495 (25.8)	562 (21.9)	2130 (26.4)	134 (21.6)
Intrauterine growth restriction n (%)	1564 (4.5)	742 (5.5)	149 (5.8)	507 (6.3)	46 (7.4)
Caesarean section n(%)	20 369 (59.0)	8099 (59.8)	1472 (57.4)	4733 (58.5)	341 (55.0)
**Maternal variables**					
Maternal ethnicity n (%)					
White	19 246 (55.8)	7885 (58.3)	1504 (58.7)	4670 (57.8)	342 (55.2)
Mixed	629 (1.8)	190 (1.4)	66 (2.6)	112 (1.4)	9 (1.5)
Asian/Asian British	3858 (11.2)	1485 (11.0)	277 (10.8)	883 (10.9)	66 (10.6)
Black African/Black Caribbean/Black British	2794 (8.1)	680 (5.0)	168 (6.6)	389 (4.8)	35 (5.6)
Other	767 (2.2)	215 (1.6)	33 (1.3)	133 (1.6)	14 (2.3)
Chorioamnionitis n (%)	5145 (14.9%)	2137 (15.8)	433 (16.9)	1331 (16.5)	107 (17.3)
Antenatal steroids given n (%)	31 553 (91.4)	12 363 (91.3)	2324 (90.7)	7356 (91.0)	555 (89.5)
IMD quintile n (%)					
1 (most deprived)	10 162 (29.4)	3993 (29.5)	934 (36.5)	2469 (30.6)	215 (34.7)
2	7742 (22.4)	2609 (19.3)	515 (20.1)	1559 (19.3)	118 (19.0)
3	5828 (16.9)	2388 (17.6)	369 (14.4)	1248 (15.4)	106 (17.1)
4	4668 (13.5)	2087 (15.4)	339 (13.2)	1150 (14.2)	86 (13.9)
5 (least deprived)	3686 (10.7)	1665 (12.3)	306 (11.9)	1055 (13.1)	82 (13.2)
**Infant postnatal variables**					
Surfactant n (%)	18 664 (54.1)	7212 (53.3)	1453 (56.7)	4494 (55.6)	447 (72.1)
Illness severity score n (%)					
0 (least ill)	14 017 (40.6)	5876 (43.4)	896 (35.0)	3240 (40.1)	110 (17.7)
1	15 359 (44.5)	5827 (43.0)	1100 (43.0)	3724 (46.1)	318 (51.3)
2	3924 (11.4)	1457 (10.8)	429 (16.8)	892 (11.0)	136 (21.9)
3 (most ill)	790 (2.3)	323 (2.4)	136 (5.3)	215 (2.7)	55 (8.9)
Transferred units in first 72 hours n (%)	4637 (13.4)	2108 (15.6)	476 (18.6)	1337 (16.5)	150 (24.2)
Level of neonatal unit on postnatal day 3 n (%)					
NICU	21 016 (60.9)	10 539 (77.9)	2561 (100)	8081 (100)	620 (100)
Local neonatal unit	12 223 (35.4)	2921 (21.6)	0 (0.0)	0 (0.0)	0 (0.0)
Special care unit	1168 (3.4)	62 (0.5)	0 (0.0)	0 (0.0)	0 (0.0)
Postnatal day of first enteral feed median (IQR)	3 (2-4)	2 (2-3)	3 (2-4)	2 (2-3)	3 (2-5)
Days to full feeds median (IQR)	13 (10–18)	13 (10–17)	13 (10–18)	13 (10–17)	22 (15–30)
Exposed to antibiotics in first three postnatal days n (%)	32 427 (94.0)	12 584 (93.0)	2463 (96.2)	7517 (93.0)	590 (95.2)

Percentage of missing data in whole cohort: caesarean section (5.2%), maternal ethnicity (21.4%), antenatal steroids (0.3%), illness severity score (0.1%), level of neonatal unit on postnatal day 3 (0.2%), postnatal day of first enteral feed (2.1%) and days to full feeds (4.6%).

IMD, index of multiple deprivation; NICU, neonatal intensive care unit.

**Figure 3:**
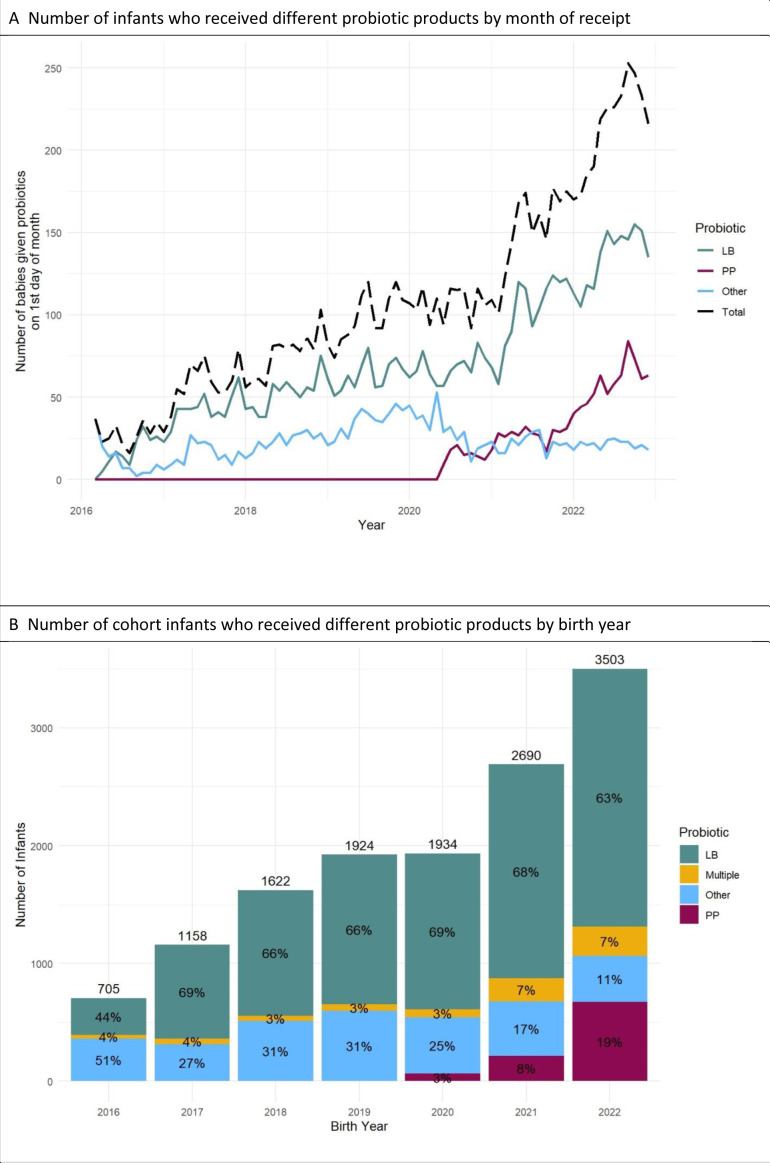
Number of infants who received each probiotic product over time. LB, Labinic; PP, Proprems.

Figure 3 shows the changes in use of particular probiotic strains over time. Prior to 2020, LB was used by the majority of units. The probiotic product PP began to be used by units in 2020 and the proportion of infants receiving this product has risen rapidly (figure 3). For the last three study months (Oct–Dec 2022), LB was still the most commonly used probiotic (629/929, ie, 67.7%), whereas 28.4% (264/929) received PP.

### Commencement and discontinuation of probiotics

**Figure 4:**
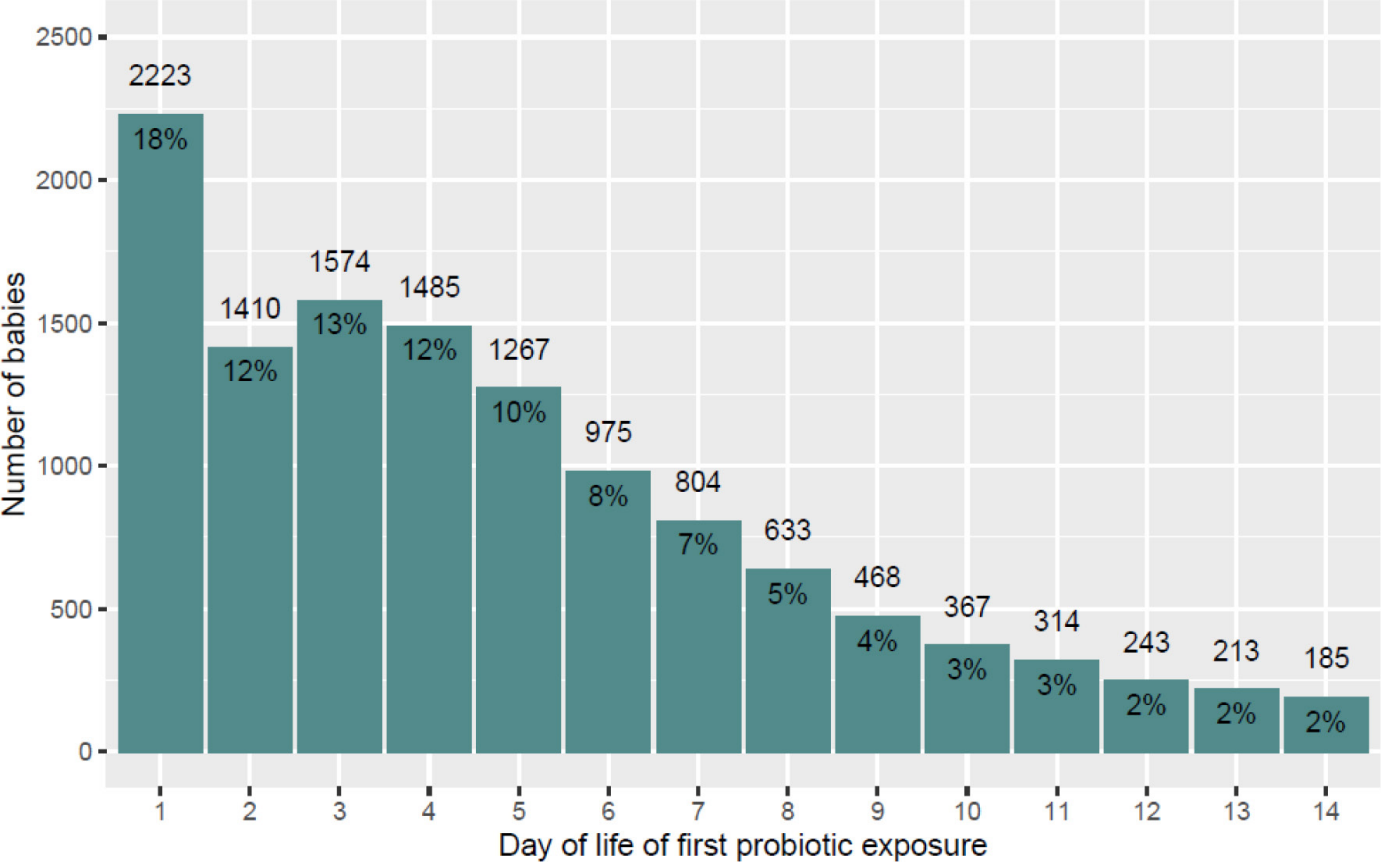
Postnatal day when probiotics are first introduced. Includes all infants who received any probiotic product in the first 14 days of life (n = 12 161). Excludes the 1375 (10.2%) of probiotic recipients who were first exposed to probiotics after day 14.

The postnatal day when probiotics were first given is shown in figure 4 (and in online supplemental figure 1 split by GA category). In probiotic-exposed infants, 71.9% (9738/13 536) received probiotics in the first week of life. Overall, probiotics were started on median day five (IQR 2–8). Probiotics were given significantly earlier (χ^2^=772.02, df=14, p<0.001) to those born at or after 28 weeks GA (median = day 4, IQR 2 to 7) compared with those born before 28 weeks GA (median = day 6, IQR 3 to 11) (online supplemental figure 1).

**Figure 5:**
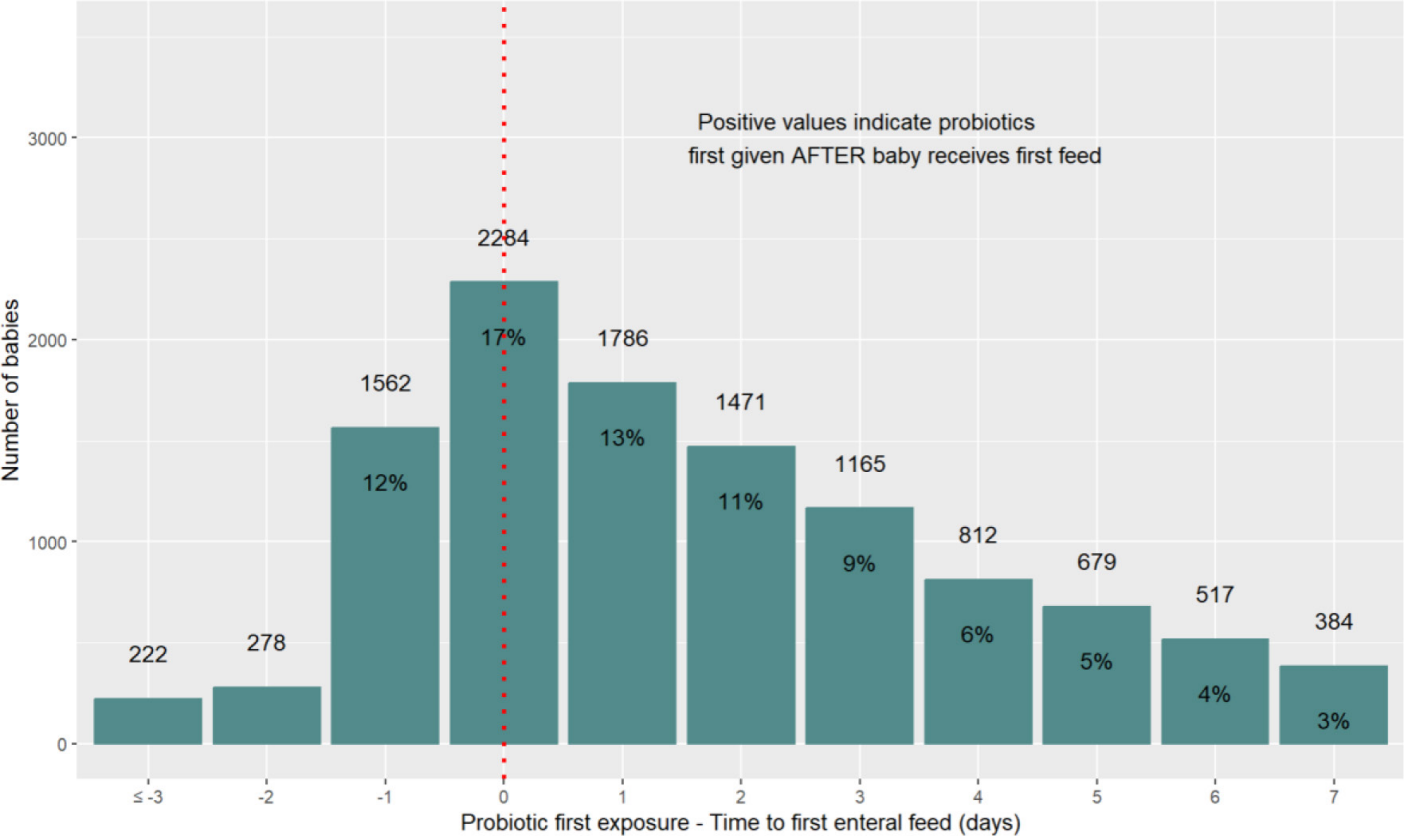
Timing of first exposure to probiotics relative to first enteral feed. 13 358 infants received any probiotic and were fed enterally. The chart excludes 2198 infants who first received probiotics more than seven days after their first enteral feed.

Figure 5 shows the relationship between the timing of the first exposure to probiotics and the first enteral feed. Probiotics were introduced at least a day before the infant received their first enteral feed in 15.4% infants (n=2062). In this group, the median interval between probiotic receipt and first feed was 1 day (IQR 1 to 1). In 17.1% infants (n=2284), probiotics were introduced on the same day as the first enteral feed. In 66.6% infants (n=9012), probiotics were introduced after the first enteral feed (median interval 4 days, IQR 2 to 7 days). 16.2% (n=2198) of infants had their first probiotic exposure more than 7 days after their first enteral feed.

In the 11 739 probiotic-exposed infants who survived to discharge, probiotics were stopped at median 33^+6^ weeks postmenstrual age (IQR 33^+0^ to 34^+2^) (online supplemental figure 2).

### Infants cared for in probiotic NICUs

Thirty of the 55 NICUs fulfilled our definition of a probiotic unit at some point during the study period. 11 262 infants (23.4% of the cohort) were cared for in probiotic NICUs on day three of life and 77.3% (8701/11 262) of these infants were exposed to probiotics. In contrast, 13.1% (4835/36 786) of infants cared for in non-probiotic units were exposed to probiotics. Online supplemental figure 3 shows the proportion of infants who received probiotics in each of the 30 probiotic NICUs over time. Table 1 presents the background characteristics of the infants treated in probiotic NICUs by timing of their first probiotic exposure.

### Factors influencing probiotic exposure in infants cared for in probiotic NICUs

In the tests of effect size, gestational age, birth weight z score and birth year each had at least one Cohen d statistic that exceeded our cut-off for inclusion in the multivariable model. Illness severity was the only categorical variable where the effect size exceeded our cut-off (Cohen’s ω=0.16 (online supplemental materials 6)).

**Figure 6:**
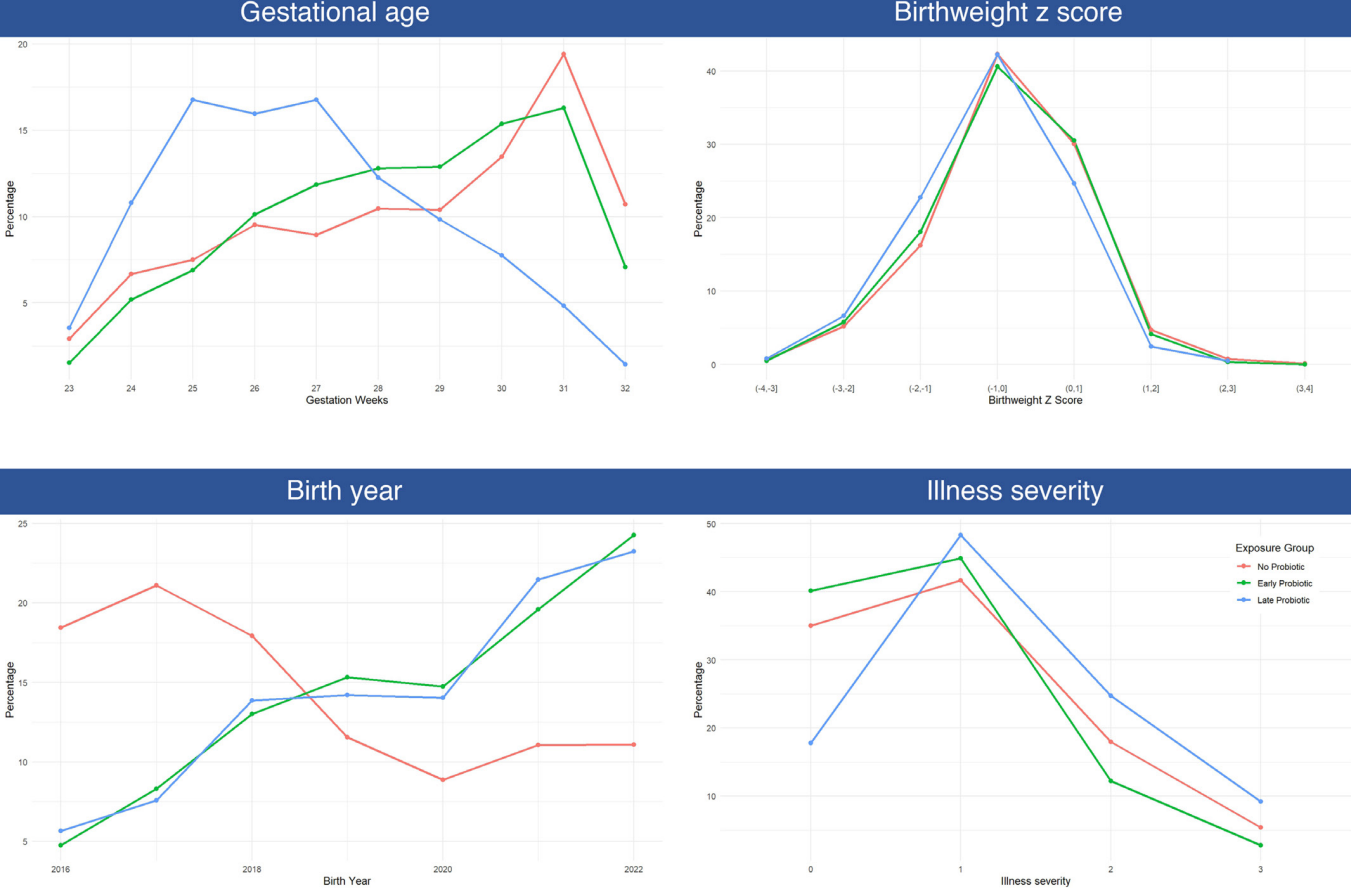
Distribution of variables associated with receipt of probiotics among infants cared for in probiotic NICUs. These categorical variables are presented as continuous for clarity of illustration.

In the multivariable analyses, these variables all showed strong evidence of an independent association with probiotic status (p<0.001 for GA, birth weight z score, birth year and illness severity). Figure 6 illustrates the gestational age, birth weight z scores, birth year and illness severity distributions of infants by probiotic exposure status. In these probiotic NICUs, infants who were exposed to probiotics were more likely to be born towards the end of the study period than infants who did not receive probiotics. Infants who first received probiotics late had lower birth weight z scores and higher illness severity scores. Infants who never received probiotics were more likely to be born either before 26 or after 30 weeks GA, compared with infants who received probiotics before day 14. However, compared with infants who received probiotics late, infants who never received probiotics were born at a later GA.

## Discussion

In this population study, we established that the proportion of preterm infants below 32 weeks GA who received probiotics in England and Wales had increased from 9% in 2016 to 54% by 2022. Sixty-five per cent of probiotic recipients received them on or before postnatal day five. By day 14, 90% of probiotic recipients had received their first probiotic. This contrasts with survey data^11^ which reported that 62/70 (89%) units who have a guideline for probiotic use aim to commence probiotics at the same time as enteral feeds. The two largest probiotics randomised controlled trials (RCTs) administered probiotics earlier, either at the point of minimal enteral feeds (ProPrems^19^) (median age of receipt day 5) or allowed the intervention before starting feeds (PiPS^20^) (median age of receipt 44 hours). Additionally, in probiotic NICUs, 23% of infants, who on the basis of their gestational age were eligible to receive probiotics, did not receive them. This finding may be surprising to those units.

We found that recipients who received probiotics late (after 14 days) had a higher illness severity score, together with lower GA and birth weight z scores than either infants who received probiotics before day 14 or those who never received them. Infants who did not receive probiotics during their neonatal stay were either born at earlier gestation with higher illness severity scores (some of whom will have died after day 4) or were more mature. The higher illness severity scores in the group who received probiotics late persist after controlling for birth year, neonatal unit providing care, birth weight z score and gestational age. This result suggests that clinicians, despite agreement at unit level to use probiotics, tend to delay them in the sickest infants. Our data also suggests that neonatal units in England and Wales that routinely use probiotics do not appear to be wholly compliant with recommendations in the ESPGHAN guidance including timing of administration and use of Good Manufacturing Practice approved products. The descriptive data shows that median time to first enteral feed is similar in infants who receive their first probiotic after day 14 compared with those who receive probiotics on or before day 14 (day 3 vs day 2). However, 25% of the group who first receive probiotics after day 14 have their first enteral feed on or after day five, and this group also reaches full feeds much later than infants who receive probiotics on or before day 14 (day 22 vs day 13). This may suggest feed intolerance contributes to delays in probiotic administration.

The principal reason for using probiotics is to reduce mortality through reductions in NEC and sepsis. While the mechanisms of action of probiotics are not well understood, the ability to stay and replicate in a host to exert benefit, the ability to improve epithelial barrier function, interference with pathogen adherence and virulence and modulation of host immunity are likely to be important. These mechanisms probably occur over several days, unlike most other therapeutic interventions that have a more rapid onset of action. In a recent review of large RCTs,^13^ the median age of NEC onset was 15.5 days postnatally, and of LOS was 18 days. Mortality that results from these events occurs later.^21^ It is therefore important in observational studies of probiotic use to explore the timing of the intervention in order to understand their ability to impact on these clinical outcomes. Most observational studies of probiotics do not do this; this may explain differences in findings^22^,^24^ following the introduction of probiotics into clinical practice. The NNRD has afforded us the opportunity to explore this question in a large population and further work is planned to address this point.

The strengths of this study include the large number of infants involved. Those infants dying in the first four days were excluded since, if they had received probiotics, it is unlikely that the probiotics would have had time to exert any benefit. We undertook careful infant classification of probiotic receipt and accounted for illness severity using key clinical interventions combined into a severity score. We suggest that this score may be useful in other settings, and further validation on an independent dataset would be valuable.

Limitations of this study include the use of routinely recorded clinical data. We cannot rule out the possibility that data were mis-recorded. However, Battersby *et al*^25^ have shown that levels of completeness and quality of data in the NNRD are high for clinical variables. The level of missing ethnicity data is concerning, but regrettably is consistent with databases from other UK health and care settings.^26^

Our finding that probiotic commencement is delayed in sicker infants is important for the interpretation and design of studies. The findings of this observational study have given us insight into what may also occur in RCTs (ie,that smaller and sicker infants are not enrolled). RCT protocols commonly exclude infants who are ‘too unstable’. In reality, this comes down to a subjective decision by the clinician and is likely to be influenced by a range of factors including the difficulty of gaining valid parental consent in the circumstance of a very sick infant and personal enthusiasm for the trial. Population level observational studies, using real world data, help us to understand factors that influence clinical practice and are important in trial design. While there is equipoise among UK clinicians about the use of probiotics, there is an opportunity to conduct observational studies on the impact of probiotic exposure. The PiPS trial^20^ demonstrated that cross-contamination between infants who do and do not receive probiotics may occur within units. Therefore, we would recommend that future observational studies perform sensitivity analyses that take account of possible cross-contamination, by assigning exposure at a unit level, rather than simply at an individual level.

The choice of probiotic product may be informed by international consensus guidance, national guidelines and other factors such as cost. Our results show that, since the introduction of the probiotic product PP in June 2020, the numbers of infants exposed to this product have been increasing (figure 3). The current situation, where two products are widely used, provides opportunities for observational studies to explore questions of relative efficacy in the prevention of NEC and probiotic sepsis, a potential risk and central concern of the US FDA.

## Conclusion

This large, population level study of probiotic use in the UK confirms that the use of probiotics has increased. In England and Wales, 54% of infants born before 32 weeks gestation in 2022 received probiotics in the first 14 days after birth. However, 23% of infants in probiotic NICUs did not receive probiotics. Our findings should help inform the design of future studies needed to address the continued uncertainty regarding the safety and efficacy of probiotics.

## Supplementary material

10.1136/bmjpo-2025-003605 online supplemental file 1

## Data Availability

Data may be obtained from a third party and are not publicly available.
