# Supplementary material for: Description of probiotic use in preterm infants in England and Wales 2016–2022
Source: BMJ Paediatr Open. 2025 Jul 24;9(1):e003605. doi: 10.1136/bmjpo-2025-003605 (PMC12306256; doi:10.1136/bmjpo-2025-003605)
Supplement: online supplemental file 1 [file bmjpo-9-1-s001.docx]

**TABLE OF CONTENTS: SUPPLEMENTARY MATERIALS AND FIGURES**

| **Supplementary materials** | |
| --- | --- |
| 1 | MAJOR CONGENITAL ABNORMALITIES |
| 2 | PROBIOTIC PRODUCT STRAINS |
| 3 | SURVEY TO ASCERTAIN PROBIOTIC STATUS OF UNITS |
| 4 | MEASURING ILLNESS SEVERITY |
| 5 | VARIABLES INVESTIGATED FOR ASSOCIATION WITH PROBIOTIC EXPOSURE IN THE POPULATION OF INFANTS TREATED IN NICUS |
| 6 | UNIVARIABLE MEASURES OF EFFECT SIZE (COHEN’S D AND COHEN’S Ω) FOR THE RELATIONSHIP BETWEEN PROBIOTIC STATUS AND BACKGROUND CHARACTERISTICS OF THE COHORT |
| 7 | ENGLISH AND WELSH MEMBERS OF UK THE NEONATAL COLLABORATIVE |
| **Supplementary figures** | |
| 1 | NUMBER AND PROPORTION OF INFANTS WHO RECEIVED PROBIOTICS BY GESTATIONAL AGE GROUP AND POSTNATAL DAY OF FIRST PROBIOTIC |
| 2 | POST-MENSTRUAL AGE WHEN PROBIOTICS ARE STOPPED |
| 3 | PROPORTION OF INFANTS RECEIVING PROBIOTICS IN PROBIOTIC NICUS OVER TIME |
| **Supplementary tables** | |
| 1 | BACKGROUND CHARACTERISTICS OF ALL PROBIOTIC RECIPIENTS STRATIFIED BY TIMING OF PROBIOTIC RECEIPT |

**SUPPLEMENTARY MATERIALS 1**

**MAJOR CONGENITAL ABNORMALITIES**

**Major congenital gastrointestinal malformations**

Correction of congenital atresia of oesophagus, oesophageal atresia, oesophageal atresia with distal tracheo-oesophageal fistula, oesophageal atresia with tracheoesophageal fistula, oesophageal atresia without distal fistula, oesophageal atresia without tracheoesophageal fistula, thoracotomy and repair of oesophageal atresia and tracheo-oesophageal fistula with primary anastomosis, atresia and stenosis of small intestine, atresia and stenosis of duodenum, duodenal atresia / stenosis / web (specify), duodenal atresia / stenosis / web, duodenal atresia / stenosis, duodenal atresia, atresia and stenosis of ileum, ileal atresia / stenosis (specify), ileal atresia / stenosis, jejunal atresia / stenosis (specify), jejunal atresia / stenosis, atresia and stenosis of large intestine, congenital absence atresia / stenosis parts of large intestine, congenital absence atresia / stenosis parts of large intestine, congenital absence atresia / stenosis of rectum with fistula, congenital absence atresia / stenosis rectum without fistula, congenital absence atresia / stenosis anus with fistula, congenital absence atresia / stenosis anus without fistula, congenital absence atresia / stenosis of large intestine part unspecified, atresia of oesophagus without fistula, atresia of oesophagus with tracheo-oesophageal fistula (tof), recurrent tracheo-oesophageal fistula, tracheo-oesophageal fistula (h-type), congenital tracheo-oesophageal fistula without atresia (tof), congenital stenosis and stricture of oesophagus, congenital stenosis of the oesophagus, congenital oesophageal web, oesophageal web, large bowel or rectum - atresia, high anorectal anomaly with rectourethral fistula, high anorectal anomaly with rectovesical fistula, high anorectal anomaly with rectocutaneous fistula, high anorectal anomaly with rectocloacal fistula, high anorectal anomaly with fistula (specify), high anorectal anomaly without fistula, anorectal anomaly - high without fistula, low anorectal anomaly with anocutaneous fistula, low anorectal anomaly with anovestibular fistula, low anorectal anomaly with fistula (other specify), congenital absence atresia / stenosis anus without fistula, anus - imperforate, imperforate anus, low anorectal anomaly without fistula, low anorectal anoma, congenital anal stenosis, persistent cloaca, exomphalos (major), exomphalos (minor), exomphalos malrotation, exomphalos, omphalocele, closure of gastroschisis includes closure of exomphalos, primary repair exomphalos, repair exomphalos using prosthesis (specify type), gastroschisis, delayed closure gastroschisis, primary repair gastroschisis, repair gastroschisis using prosthesis (specify type), silo insertion for reduction of gastroschisis, delayed closure exomphalos, cutback of covered anus, repair of imperforate anus (with or without vaginal, cutback of low anorectal anomaly (nixon), oesophageal atresia - repair of anastomotic leak, primary repair of oesophageal atresia, closure of recurrent tracheo-oesophageal fistula, closure of tracheooesophageal fistula, closure of tracheo-oesophageal fistula, duodenal atresia/stenosis repair, duodenal atresia/stenosis repair (von)

**Other severe congenital conditions, lethal or requiring early surgical intervention**

*Cardiac and circulatory system*

Congenital malformations of cardiac chambers and connections, common arterial trunk (truncus malformation), truncus arteriosus, double outlet right ventricle (dorv), double outlet left ventricle (dolv), dextrotransposition of aorta, transposition great arteries (tga), transposition of the great vessels (tga), double inlet ventricle (dilv), discordant atrioventricular connection, isomerism of atrial appendages, atrial isomerism & asplenia, atrial isomerism with asplenia, atrial isomerism with polyspenia, atrial isomerism, other congenital malforms of cardiac chambers and connections, congenital malforms of cardiac chambers and connections unspec, complete atrioventricular septal defect, atrio-ventricular septal defect (avsd), atrioventricular septal defect (avsd), tetralogy of fallot, atrium single, ventricle single, congenital malformations of pulmonary and tricuspid valves, pulmonary valve atresia, congenital pulmonary valve stenosis, pulmonary valve stenosis (ps), congenital pulmonary valve insufficiency, other congenital malformations of pulmonary valve, congenital tricuspid atresia / stenosis, ebstein's anomaly, hypoplastic right heart syndrome, other congenital malformations of tricuspid valve, congenital malformation of tricuspid valve (unknown or unspecified cause), congenital malformations of aortic and mitral valves, congenital stenosis of aortic valve (as), bicuspid aortic valve, mitral atresia, congenital insufficiency of aortic valve, congenital mitral stenosis (ms), hypoplastic left heart syndrome (hlh), other congenital malformations of aortic and mitral valves, congenital malformation of aortic and mitral valves unspec, coarctation of aorta, coarctation of the aorta, stenosis of aorta (as), other malformation of aorta, malformation of aorta, double aortic arch, hypoplasia of aortic arch, interrupted aortic arch, atresia of pulmonary artery, pulmonary stenosis (physiological branch stenosis), pulmonary stenosis - branch, other congenital malformations of great arteries, total anomalous pulmonary venous connection (tapvd), total anomylous pulmonary venous drainage (tapvd), blalock-taussig shunt

*Respiratory system, including diaphragmatic hernia*

Choanal atresia - bilateral, choanal atresia - unilateral (l), choanal atresia - unilateral (r), choanal atresia / stenosis (specify), choanal stenosis, congenital malformations of trachea and bronchus, congenital tracheomalacia, tracheomalacia, other congenital malformations of trachea, tracheal agenesis or atresia, bronchomalacia, congenital malformations of bronchus, congenital cystic lung (ccam), congenital cystic lung (congenital lobar emphysema), congenital cystic lung, sequestration of lung, congenital bronchiectasis, hypoplasia and dysplasia of lung, repair choanal atresia, congenital diaphragmatic hernia, congenital diaphragic hernia, morgagni diaphragmatic hernia, diaphragmatic hernia - left, diaphragmatic hernia - right, recurrent congenital diaphragmatic hernia, eventration of diaphragic hernia, eventration of the diaphragm, repair of congenital diaphragmatic hernia, prosthetic repair of congenital diaphragmatic hernia (specify), aplasia of the diaphragm, fetoscopic insertion of tracheal plug for congenital diaphragmatic hernia, other repair of diaphragmatic hernia (specify), other specified repair of diaphragmatic hernia, repair of diaphragmatic hernia using abdominal approach nec, primary repair of congenital diaphragmatic hernia, thoracoscopic repair of congenital diaphragmatic

*Brain and nervous system*

Does not include spina bifida occulta

Frontal encephalocele, nasofrontal encephalocele, occipital encephalocele, encephalocoele - occipital, encephalocele (unknown or unspecified cause), encephalocele, meningocele (specify site), myelomeningocele (specify site), meningocele & hydrocephalus (specify site), thoracic spina bifida with hydrocephalus, lumbar spina bifida with hydrocephalus, sacral spina bifida with hydrocephalus, (unknown or unspecified cause) spina bifida with hydrocephalus, cervical spina bifida without hydrocephalus, thoracic spina bifida without hydrocephalus, lumbar spina bifida without hydrocephalus, sacral spina bifida without hydrocephalus, spina bifida (unknown or unspecified cause), spina bifida, repair of spina bifida, repair of encephalocele, anencephaly and similar malformations, anencephaly, craniorachischisis, iniencephaly, holoprosencephaly, closure of spinal myelomeningocele, closure of spinal meningocele

*Urinary system*

Bilateral renal agenesis, renal agenesis, bilateral, potter's syndrome, autosomal recessive polycystic kidney - infantile, polycystic kidney, infantile type, autosomal dominant polycystic kidney in childhood, polycystic kidney, adult type, polycystic kidney, exstrophy of urinary bladder, bladder exstrophy, posterior urethral valves (puv), congenital posterior urethral valves (puv), congenital absence of bladder and urethra

*Other miscellaneous lethal conditions*

Thanatophoric short stature, edwards syndrome (trisomy 18), Edwards syndrome (unknown or unspecified cause), trisomy 18, Patau syndrome (trisomy 13), trisomy 13, sirenomelia, triploidy and polyploidy

**SUPPLEMENTARY MATERIALS 2**

**PROBIOTIC PRODUCT STRAINS**

| **Product Name** | **Probiotic Strains** |
| --- | --- |
| Labinic and LB2 | *Lactobacillus acidophilus, Bifidobacterum bifidum* and *Bifidobacterium infantis* |
| Proprems | *Bifidobacterium infantis Bb-02 (DSM 33361), Bifidobacterium lactis (BB-12®)* and *Streptococcus thermophilus (TH-4®)* |
| Bio-kult | *Lactobacillus paracasei PXN® 37™, Lactobacillus rhamnosus PXN® 54™, Streptococcus thermophilus PXN® 66™, Lactobacillus helveticus PXN® 35™, Bifidobacterium breve PXN® 25™, Bifidobacterium infantis PXN® 27™* and *Lactobacillus delbrueckii ssp. bulgaricus PXN® 39™* |
| Infloran | *Bifidobacterium bifidum NCDO 2203* and *Lactobacillus acidophilus NCDO 1784* |

**SUPPLEMENTARY MATERIALS 3**

**SURVEY TO ASCERTAIN PROBIOTIC STATUS OF UNITS**

**Selection of units to be surveyed**

We had originally identified units as probiotic units if there was at least one study month where a majority of infants, who were cared for in the unit on day 3 of life, received probiotics in the first 14 days of life. We identified 31 units where:

- there was a mismatch between their probiotic status based on the NNRD data and their response to the NeoTRIPS survey (1) (20 units)
- the unit had not responded to the NeoTRIPS survey or was not surveyed by NeoTRIPS (9 units)
- the unit had responded to the NeoTRIPS survey to say they routinely used probiotics and they met the definition of a probiotic unit for at least one study month, but it was unclear from the NNRD data when the probiotic policy had been introduced (2 units)

**Contents of the survey**

We surveyed these 31 units about their usage of probiotics via email.

In the survey we asked units:

1. Whether they routinely used probiotics

2. If they routinely used probiotics, when did they introduce the probiotic care guideline

3. What the criteria were for an infant to receive probiotics e.g. weight, gestational age.

**Results of the survey**

20 units (65) responded to the survey and one unit had closed before the survey began. 18 of those 20 units confirmed that they routinely used probiotics. Two units stated that they did not routinely use probiotics, although one of these two units would continue probiotics if an infant was transferred to their unit and had been given probiotics at their previous unit.

**Recoding in response to survey results**

One unit who did not respond to our survey, stated in the NeoTRIPS survey that they did not use probiotics. Based on NNRD data we had originally categorised this unit as a probiotic unit for 5 study months. We recoded this unit as a non-probiotic unit, given their response to the NeoTRIPS survey. As a result, 59 infants were recoded as cared for in a non-probiotic unit.

In response to the survey results, the unit status of 2,508 infants were amended and the number of infants cared for in probiotic units rose to 15,680.

**SUPPLEMENTARY MATERIALS 4**

**MEASURING ILLNESS SEVERITY**

**Illness severity score**

To help control for confounding by indication, we created a score of illness severity in the first two days of life. Receiving any one of inotropes, invasive respiratory support or nitric oxide, in the first two days of life, added one to the illness severity score. Illness severity was therefore scored from zero to three, where three represented the most ill infants who received inotropes, invasive respiratory support and nitric oxide, and zero represented the least ill infants who did not receive any of inotropes, invasive respiratory support or nitric oxide.

**Distribution of illness severity scores**

| **Illness severity score** | **Percentage of cohort** |
| --- | --- |
| 0 | 41.8% |
| 1 | 44.5% |
| 2 | 11.3% |
| 3 | 2.3% |

This illness severity score is unvalidated. However the score is associated with infant mortality after day 4 of life in the study cohort, χ²(df = 4, N = 47,573) = 2,522.1, p < .001. A likelihood ratio test showed evidence of a non-linear trend in the association.


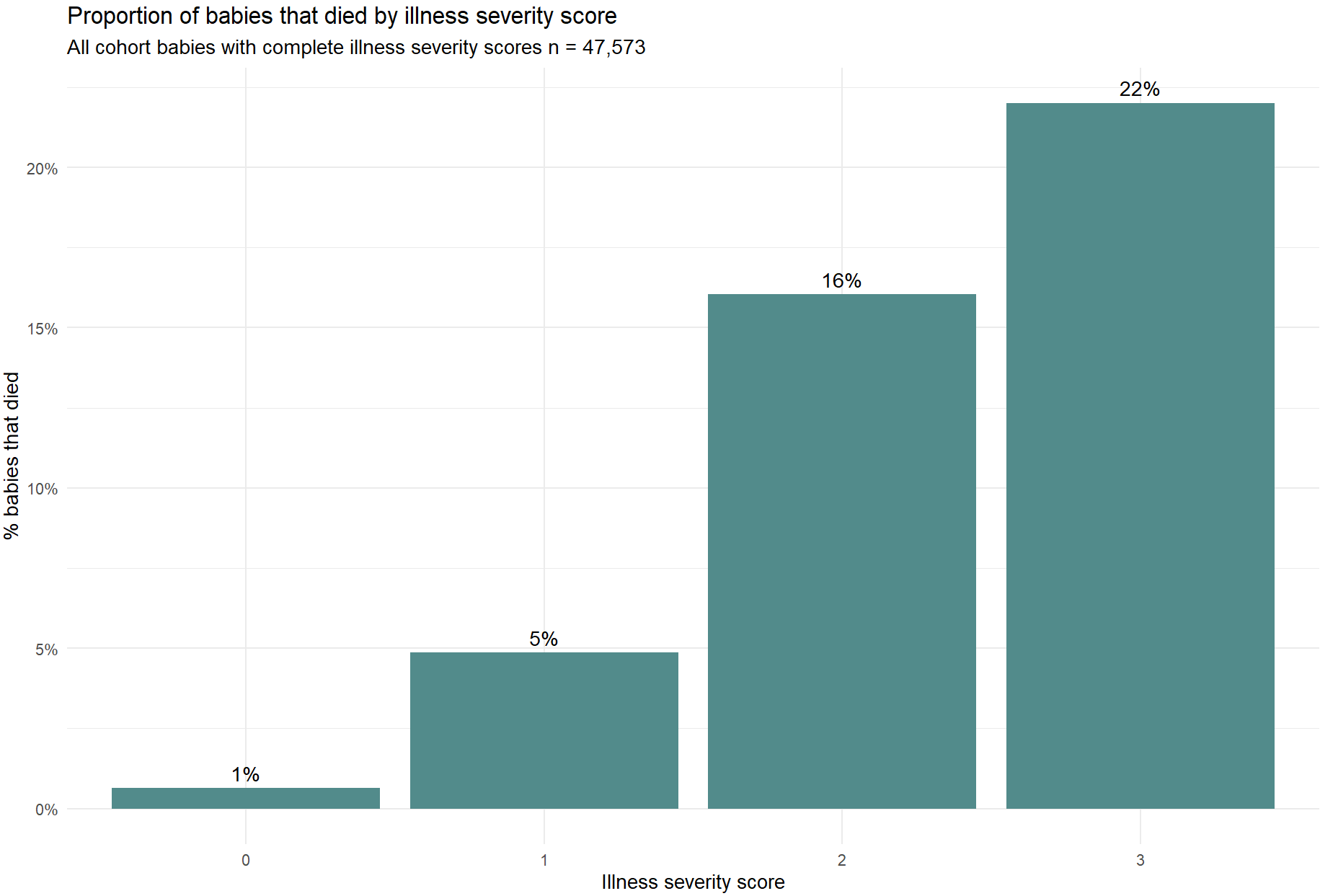


**SUPPLEMENTARY MATERIALS 5**

**VARIABLES INVESTIGATED FOR ASSOCIATION WITH PROBIOTIC EXPOSURE IN THE POPULATION OF INFANTS TREATED IN NICUS**

| **Variable** | **Variable type** | **Details** |
| --- | --- | --- |
| **Infant background variables** | | |
| **Sex** | Three level nominal | Coded as male, female, indeterminate |
| **Gestational age** | Continuous | Measured in days |
| **Birthweight z score** | Continuous |  |
| **Birth year** | Continuous |  |
| **Multiple birth** | Binary |  |
| **Intra-uterine growth restriction** | Binary | Coded as IUGR present if birthweight-for-age z-score <-2SD. Derived from birthweight z-score calculated against reference cohort (2) |
| **Elective caesarean section** | Binary |  |
| **Emergency caesarean section** | Binary |  |
|  | | |
| **Maternal variables** | | |
| **Ethnicity** | Six level nominal | Coded as White, Mixed, Asian/Asian British, Black African/Black Caribbean/Black British, Other and Missing. |
| **Gravidity** | Interval |  |
| **Maternal infection** | Binary | Coded as 1 if any of maternal pyrexia in labour> 38⁰C, intrapartum antibiotics given or maternal diagnosis of chorioamnionitis are coded as ‘Yes’ in NNRD |
| **Chorioamnionitis** | Binary |  |
| **Antenatal steroids** | Binary | Coded as any antenatal steroids given during pregnancy (partial or complete course) =1; No antenatal steroids given=0. |
| **Index of multiple deprivation** | Five level ordinal | A broad measure of deprivation based on the mother’s reported postcode at time of birth, analysed as quintiles (3). Derived from mother’s Lower Level Super Output Area. |
|  | | |
| **Infant postnatal variables** | | |
| **Surfactant** | Binary | Coded as 1 if surfactant is given either in the delivery room or on the neonatal unit on the day of birth |
| **Illness severity score** | Four level ordinal | Illness severity score derived from the sum of:  1. Did the infant receive inotropes on either day 1 or 2 (coded as 1 or 0) (NNRD field: InotropesGiven OR any of the following drugs listed in the DrugsDay field: Adrenaline, Dopamine, Dobutamine, Milrinone, Noradrenaline or Vasopressin)  2. Did the infant receive any invasive respiratory support on either day 1 or 2 (coded as 1 or 0)  3. Did the infant receive any nitric oxide on either day 1 or 2 (coded as 1 or 0) |
| **Fed enterally in the first four days** | Binary |  |
| **Level of care** | Three level ordinal | Maximum level of care received by the infant in the first four days of life using the British Association of Perinatal Medicine categorisation (4) coded as: Intensive care, High dependency care or Special Care/Normal. |
| **Transferred units in first 72 hours** | Binary |  |

**SUPPLEMENTARY MATERIALS 6**

**UNIVARIABLE MEASURES OF EFFECT SIZE FOR THE RELATIONSHIP BETWEEN PROBIOTIC STATUS AND BACKGROUND CHARACTERISTICS OF THE COHORT**

1. **Continuous background variables**

| **Variable** | **Mean difference** | | | **Cohens d** | | |
| --- | --- | --- | --- | --- | --- | --- |
|  | **Never exposed vs Exposed at or before day 14** | **Never exposed vs Exposed after day 14** | **Exposed at or before day 14**  **vs Exposed after day 14** | **Never exposed vs Exposed at or before day 14** | **Never exposed vs Exposed after day 14** | **Exposed at or before day 14**  **vs Exposed after day 14** |
| Gestational age (weeks) | 0.06 | 1.60 | 1.54 | 0.03 | 0.64 | 0.67 |
| Birthweight z score | 0.06 | 0.21 | 0.15 | 0.06 | 0.22 | 0.16 |
| Birth year (years) | -1.34 | -1.32 | 0.02 | 0.72 | 0.67 | 0.01 |

1. **Binary background variables**

| **Variable** | **Odds Ratio (95% CI)** | | | **Cohen’s ω** |
| --- | --- | --- | --- | --- |
|  | **Never exposed vs Exposed at or before day 14** | **Never exposed vs Exposed after day 14** | **Exposed at or before day 14**  **vs Exposed after day 14** |  |
| **Infant background variables** | | | | |
| Multiple birth | 0.79 (0.71-0.87) | 1.02 (0.83-1.27) | 1.30 (1.07-1.59) | 0.05 |
| Intra-uterine growth restriction | 0.92 (0.76-1.11) | 0.77 (0.55-1.10) | 0.84 (0.62-1.16) | 0.01 |
| Elective caesarean section | 0.78 (0.64-0.95) | 1.11 (0.75-1.73) | 1.42 (0.98-2.15) | 0.03 |
| Emergency caesarean section | 1.01 (0.92-1.10) | 1.07 (0.89-1.29) | 1.07 (0.90-1.27) | 0.01 |
| **Maternal variables** | | | | |
| Maternal infection | 0.95 (0.87-1.05) | 1.03 (0.85-1.25) | 1.08 (0.91-1.29) | 0.01 |
| Chorioamnionitis | 1.03 (0.92-1.16) | 0.98 (0.78-1.24) | 0.95 (0.77-1.18) | 0.01 |
| Antenatal steroids | 0.98 (0.84-1.14) | 1.18 (0.88-1.57) | 1.21 (0.92-1.57) | 0.01 |
| **Infant postnatal variables** | | | | |
| Surfactant | 1.05 (0.96-1.14) | 0.51 (0.42-0.61) | 0.49 (0.40-0.58) | 0.08 |
| Fed enterally in the first four days | 1.11 (0.93-1.32) | 0.60 (0.45-0.81) | 0.54 (0.42-0.71) | 0.04 |
| Transferred units in first 72 hours | 1.15 (1.03-1.29) | 0.72 (0.58-0.88) | 0.62 (0.51-0.76) | 0.05 |

1. **Non-binary categorical background variables**

| **Variable** | **Variable type** | **Χ^2^ statistic (df)** | **Measure of effect size**  **(Cohen’s ω)** |
| --- | --- | --- | --- |
| **Infant background variables** | | | |
| Sex | Nominal | 7.2 (4) | 0.03 |
| **Maternal variables** | | | |
| Ethnicity | Nominal | 199.0 (10) | 0.06 |
| Gravidity | Ordinal | 30.6 (8) | 0.06 |
| Index of multiple deprivation | Ordinal | 96.1 (8) | 0.05 |
| **Infant postnatal variables** | | | |
| Illness severity score | Ordinal | 463 (6) | 0.16 |
| Level of care | Ordinal | 358 (4) | 0.08 |

**SUPPLEMENTARY MATERIALS 7**

**ENGLISH AND WELSH MEMBERS OF THE UK NEONATAL COLLABORATIVE**

**Institution Lead**

Airedale General Hospital Dr Matthew Babirecki

Alder Hey Dr Rebecca Kettle

Arrowe Park Hospital Dr Anand Kamalanathan

Barnet Hospital Dr Clare Cane

Barnsley District General Hospital Dr Kavi Aucharaz

Basildon Hospital Dr Rathod Poorva

Basingstoke & North Hampshire Hospital Dr Jummy Awoseyila

Bassetlaw District General Hospital Dr L M Wong

Bedford Hospital Dr Anita Mittal

Birmingham City Hospital Dr Penny Broggio

Birmingham Heartlands Hospital Dr Pinki Surana

Birmingham Women's Hospital Dr Matt Nash

Bradford Royal Infirmary Dr Sam Wallis

Broomfield Hospital, Chelmsford Dr Ahmed Hassan

Calderdale Royal Hospital Dr Karin Schwarz

Chelsea & Westminster Hospital Dr Shu-Ling Chuang

Chesterfield & North Derbyshire Royal Hospital Dr Penelope Young

Colchester General Hospital Dr Ramona Onita

Conquest Hospital Dr Mani Kandasamy

Countess of Chester Hospital Dr Stephen Brearey

Croydon University Hospital Dr Joselyn Morris

Cumberland Infirmary Dr Rachel Smith

Darent Valley Hospital Dr Bharath Gowda

Darlington Memorial Hospital Dr Mehdi Garbash

Derriford Hospital Dr Alex Allwood

Diana Princess of Wales Hospital Dr Vijaya Hebbar

Doncaster Royal Infirmary Dr Nigel Brooke

Dorset County Hospital Dr Claire Hollinsworh

East Surrey Hospital Dr Toria Klutse

Epsom General Hospital Dr Clare Sturdy

Frimley Park Hospital Dr Sathish Krishnan

Furness General Hospital Dr Maria Hadjicosta

George Eliot Hospital Dr Sabyasachi Chowdhury

Gloucester Royal Hospital Dr Shyam Bhakthavalsala

Good Hope Hospital Dr Daniel Dogar

Great Western Hospital Dr Girish Gowda

Guy's & St Thomas' Hospital Dr Karen Turnock

Harrogate District Hospital Dr Patricia Gilbertson

Hereford County Hospital Dr Cath Seagrave

Hillingdon Hospital Dr Tristan Bate

Hinchingbrooke Hospital Dr Hilary Dixon

Homerton Hospital Dr Narendra Aladangady

Hull Royal infirmary Dr Hassan Gaili

Ipswich Hospital Dr Prathiba Pai

James Cook University Hospital Dr M Lal

James Paget Hospital Dr Oluseun Tayo

Kettering General Hospital Dr Abraham Isaac

Kings College Hospital Dr Carolina Zorro

King's Mill Hospital Dr Dhaval Dave

Kingston Hospital Dr Jonathan Filkin

Lancashire Women and Newborn Centre Dr Savi Sivashankar

Leeds General Infirmary Dr Hannah Shore

Leicester General Hospital Dr Jo Behrsin

Leicester Royal Infirmary Dr Jo Behrsin

Leighton Hospital Dr Michael Grosdenier

Lincoln County Hospital Dr Ruchika Gupta

Lister Hospital Dr Ather Ahmed

Liverpool Women's Hospital Dr Nim Subhedar

Luton & Dunstable Hospital Dr Jennifer Birch

Macclesfield District General Hospital Dr Surendran Chandrasekaran

Manor Hospital (Walsall) Dr Ashok Karupaiah

Medway Maritime Hospital Dr Ghada Ramadan

Milton Keynes General Hospital Dr I Misra

Musgrove Park Hospital Dr Chris Knight

New Cross Hospital Dr Richard Heaver

Newham General Hospital Dr Mohammad Alam

Nobles Hospital Dr Prakash Thiagarajan

Norfolk & Norwich University Hospital Dr Florence Walston

North Devon District Hospital Dr Tiziana Fragapane

North Manchester General Hospital Dr Bivan Saha

North Middlesex University Hospital Dr Cheentan Singh

Northampton General Hospital Dr Nick Barnes

Northumbria Specialist Emergency Care Hospital  Dr Sangeeta Tiwary

Northwick Park Hospital Dr Richard Nicholl

Nottingham City Hospital Dr Dush Batra

Nottingham University Hospital (QMC) Dr Dush Batra

Ormskirk District General Hospital Dr Victoria Nesbitt

Oxford University Hospitals, John Radcliffe Hospital Dr Amit Gupta

Peterborough City Hospital Dr Katharine McDevitt

Pilgrim Hospital Dr Ruchika Gupta

Pinderfields General Hospital Dr David Gibson

Poole General Hospital Dr Peter Mcewan

Princess Alexandra Hospital Dr Sanath Reddy

Princess Anne Hospital Dr Mark Johnson

Princess Royal Hospital Dr Cassie Lawn

Princess Royal Hospital Telford Dr Alison Belfitt and Dr Jennifer Brindley

Princess Royal University Hospital Dr Rashmi Gandhi

Queen Alexandra Hospital Dr Charlotte Groves

Queen Charlotte's Hospital Dr Aniko Deierl

Queen Elizabeth Hospital, Gateshead Dr Shilpa Ramesh

Queen Elizabeth Hospital, King's Lynn Dr Salamatu Jalloh

Queen Elizabeth Hospital, Woolwich Dr Julia Croft

Queen Elizabeth the Queen Mother Hospital Dr Bushra Abdul-Malik

Queen's Hospital, Burton on Trent Dr Dominic Muogbo

Queen's Hospital, Romford Dr Ambalika Das

Queen's Hospital, Romford 2 Dr Khalid Mannan

Rosie Maternity Hospital, Addenbrookes Dr Shazia Hoodbhoy

Rotherham District General Hospital Dr Soma Sengupta

Royal Albert Edward Infirmary Dr Christos Zipitis

Royal Berkshire Hospital Dr Kemy Naidoo

Royal Bolton Hospital Dr Dinakar Seshadri

Royal Cornwall Hospital Dr Chris Warren

Royal Derby Hospital Dr Nigel Ruggins

Royal Devon & Exeter Hospital Dr Chrissie Oliver

Royal Hampshire County Hospital Dr Lucinda Winckworth

Royal Lancaster Infirmary Dr Joanne Fedee

Royal Oldham Hospital Dr Anitha Vayalakkad

Royal Preston Hospital Dr Richa Gupta

Royal Stoke University Hospital Dr Julia Uffindell

Royal Surrey County Hospital Dr Jo MacLeod

Royal Sussex County Hospital Dr Cassie Lawn

Royal United Hospital Dr Rebecca Winterson

Royal Victoria Infirmary Dr Naveen Athiraman

Russells Hall Hospital Dr Muhammad Khurshid

Salisbury District Hospital Dr Jim Baird

Scarborough General Hospital Dr Adedayo Owoeye

Scunthorpe General Hospital Dr Umapathee Majuran

Sheffield Children's Hospital Dr Richard Lindley

Southend Hospital Dr Vineet Gupta

Southmead Hospital Dr Faith Emery and Dr Madhavi Parvathareddy

St George's Hospital Dr Donovan Duffy

St Helier Hospital Dr Salim Yasin

St James University Hospital Dr Hannah Shore

St Mary's Hospital, IOW Dr Akinsola Ogundiya

St Mary's Hospital, London Dr Aniko Deierl

St Mary's Hospital, Manchester Dr Arin Mukherjee

St Michael's Hospital Dr Pamela Cairns

St Peter's Hospital Dr Vennila Ponnusamy

St Richard's Hospital Dr Victoria Sharp

Stepping Hill Hospital Dr Carrie Heal

Stoke Mandeville Hospital Dr Sanjay Salgia

Sunderland Royal Hospital Dr Imran Ahmed

Tameside General Hospital Dr Helen Purves

The Jessop Wing, Sheffield Dr Porus Bastani

The Royal Free Hospital Dr Eleanor Bond

The Royal London Hospital - Constance Green Dr Divyen Shah

Torbay Hospital Dr Esther Morris

Tunbridge Wells Hospital  Dr Mithun Urs

University College Hospital Dr Giles Kendall

University Hospital Coventry Dr Puneet Nath

University Hospital Lewisham Dr Igor Fierens

University Hospital of North Durham Dr Mehdi Garbash

University Hospital of North Tees Dr Hari Kumar

Victoria Hospital, Blackpool Dr Peter Curtis

Warrington Hospital Dr Delyth Webb

Warwick Hospital Dr Sumedha Bird

Watford General Hospital Dr Sankara Narayanan

West Cumberland Hospital Dr Yee Mon Aung

West Middlesex University Hospital Dr Elizabeth Eyre

West Suffolk Hospital Dr Tayyaba Aamir

Wexham Park Hospital Dr Angela Yannoulias

Whipps Cross University Hospital Dr Caroline Sullivan

Whiston Hospital Dr Ros Garr

Whittington Hospital Dr Wynne Leith

William Harvey Hospital Dr Shaveta Mulla

Worcestershire Royal Hospital Dr Anna Gregory

Worthing Hospital Dr Edward Yates

Wythenshawe Hospital  Dr Abijeet Godhamgaonkar

Yeovil District Hospital Dr Siba Paul

York District Hospital Dr Sundeep Sandhu

Singleton Hospital Dr Arun Ramachandran

Princess of Wales Hospital Dr Abby Parish

The Grange University Hospital Dr Anitha James

Glan Clwyd Hospital Dr Ambrose Onibere

Wrexham Maelor Hospital Dr Artur Abelian

Ysbyty Gwynedd Dr Shakir Saeed

University Hospital of Wales Dr Nitin Goel

Prince Charles Hospital Dr Shikha Jain

Glangwili General Hospital Dr Prem Pitchaikani

**SUPPLEMENTARY FIGURE 1**

**NUMBER AND PROPORTION OF INFANTS WHO RECEIVED PROBIOTICS BY GESTATIONAL AGE GROUP AND POSTNATAL DAY OF FIRST PROBIOTIC**

Totals and percentages are based on all infants that receive probiotics at any time. Chart excludes 483 infants born before 28 weeks and 892 infants born at or after 28 weeks, who first received probiotics more than 14 days after birth.


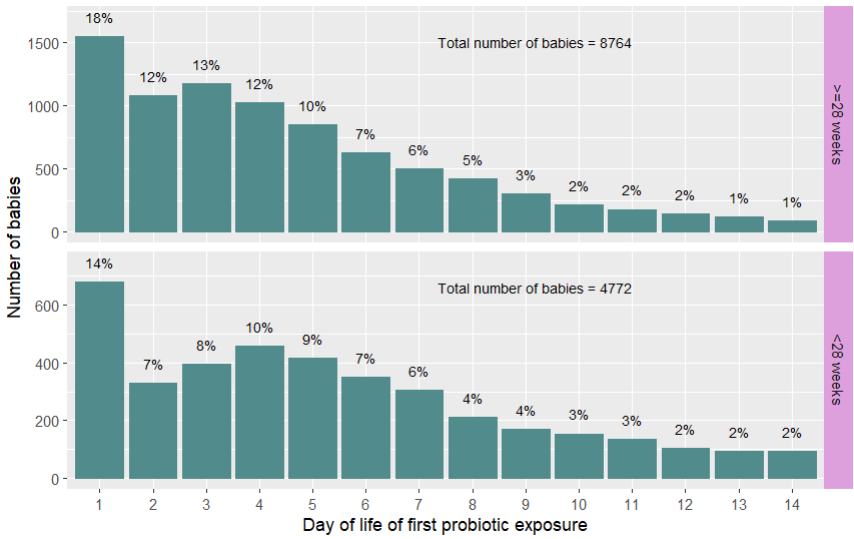


**SUPPLEMENTARY FIGURE 2**

**POST-MENSTRUAL AGE WHEN PROBIOTICS ARE STOPPED**

Includes all infants who received any probiotic in the first 14 days of life and survived to discharge (n=13,037)


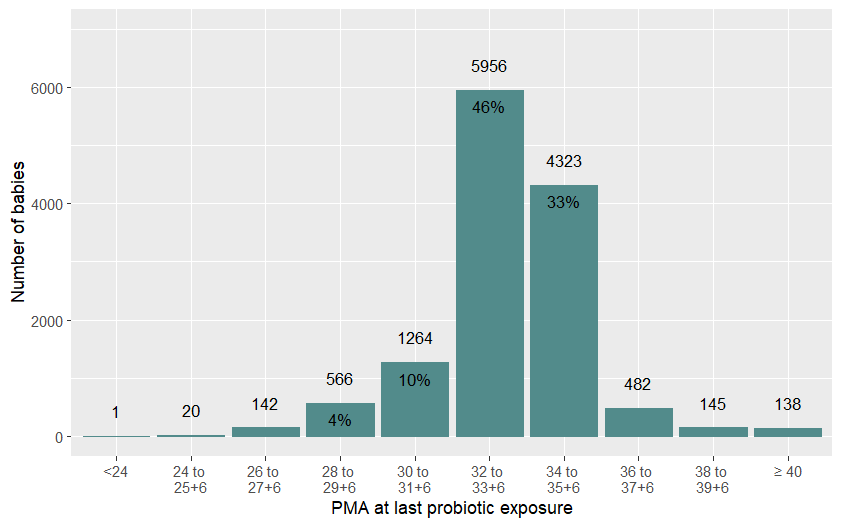


**SUPPLEMENTARY FIGURE 3**

**PROPORTION OF INFANTS RECEIVING PROBIOTICS IN PROBIOTIC NICUS OVER TIME**


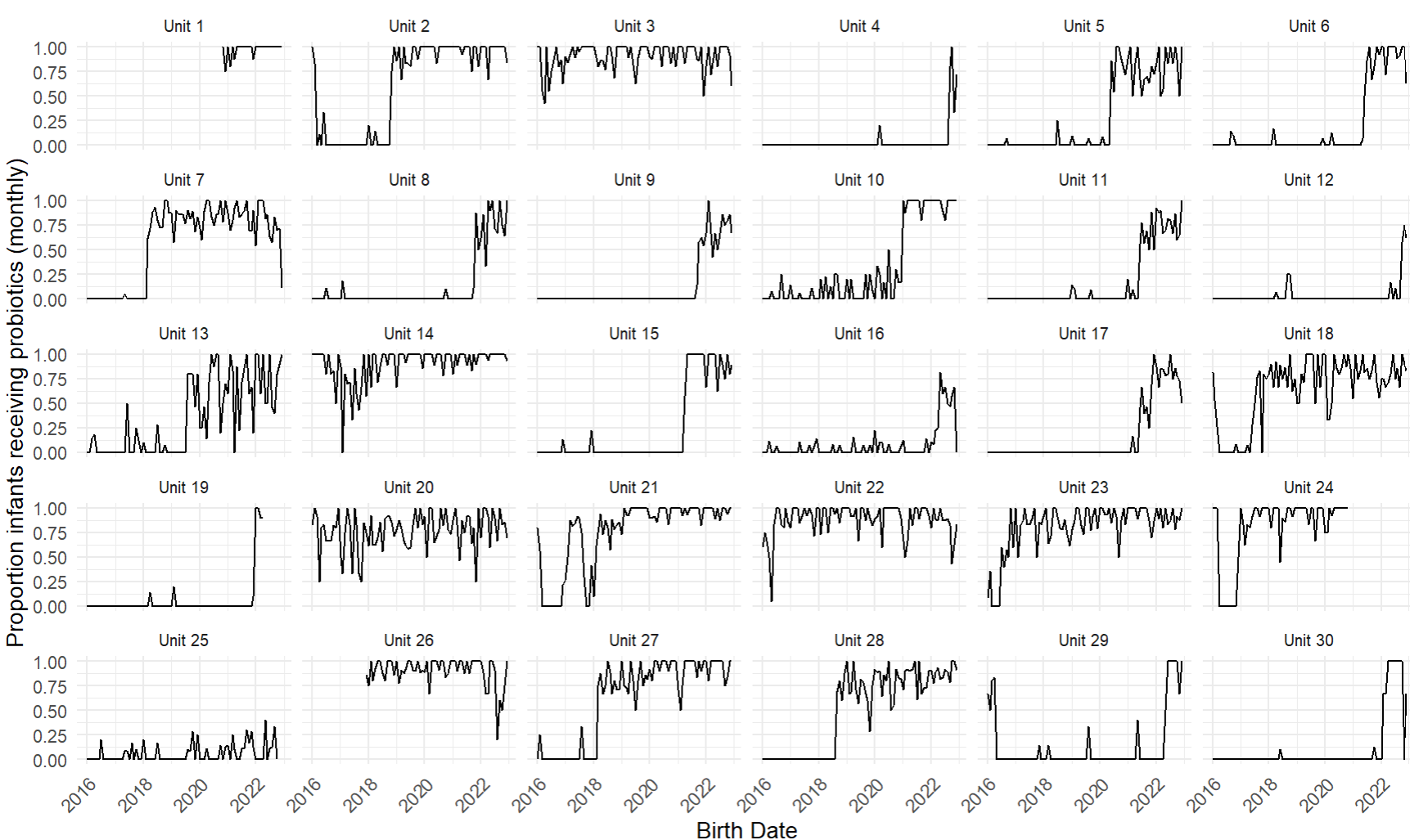


**SUPPLEMENTARY TABLE 1**

**BACKGROUND CHARACTERISTICS OF ALL PROBIOTIC RECIPIENTS STRATIFIED BY TIMING OF PROBIOTIC RECEIPT**

|  | | | **All probiotic recipients (n= 13,536)** | | | |
| --- | --- | --- | --- | --- | --- | --- |
|  |  |  | **First exposed**  **≤ day 14** | | **First exposed**  **> day 14** | |
| Number of infants (%) | | | 12,161 (89.8%) | | 1,375 (10.2%) | |
| **Infant background variables** | | | | | | |
| Male Sex n (%) | | | 6,684 (55.0) | | 722 (52.5) | |
| Gestational age at birth Median (IQR) | | | 29.3 (27.3-30.7) | | 26.9 (25.3-28.7) | |
| Birthweight (grams) Median (IQR) | | | 1,170 (900-1,460) | | 852 (692-1,100) | |
| Birth year  n (%) | | 2016-2019 | 4,756 (39.1) | | 653 (47.5) | |
|  |  | 2020-2022 | 7,405 (60.9) | | 722 (52.5) | |
| Multiple birth n(%) | | | 3,162 (26.0) | | 333 (24.2) | |
| Intrauterine growth restriction n(%) | | | 646 (5.3) | | 96 (7.0) | |
| Caesarean section n (%) | | | 7,354 (60.5) | | 745 (54.2) | |
| **Maternal variables** | | | | | | |
| Maternal ethnicity  n (%) | | White | 7,141 (58.7) | 744 (54.1) | |  |
|  |  | Mixed | 174 (1.4) | 16 (1.2) | |  |
|  |  | Asian / Asian British | 1,315 (10.8) | 170 (12.4) | |  |
|  |  | Black African / Black Caribbean / Black British | 584 (4.8) | 96 (7.0) | |  |
|  |  | Other | 190 (1.6) | 25 (1.8) | |  |
| Chorioamnionitis n (%) | | | 10,262 (84.4) | 1,137 (82.7) | |  |
| Antenatal steroids given n (%) | | | 11,109 (91.3) | 1,254 (91.2) | |  |
| IMD quintile  n (%) | | 1 (most deprived) | 3,556 (29.2) | 437 (31.8) | |  |
|  |  | 2 | 2,332 (19.2) | 277 (20.1) | |  |
|  |  | 3 | 2,138 (17.6) | 250 (18.2) | |  |
|  |  | 4 | 1,879 (15.5) | 208 (15.1) | |  |
|  |  | 5 (least deprived) | 1,499 (12.3) | 166 (12.1) | |  |
| **Infant postnatal variables** | | | | | | |
| Surfactant given n (%) | | | 6,207 (51.0) | 1,005 (73.1) | |  |
| Illness severity score n (%) | 0 (least ill) | | 5,595 (46.0) | 281 (20.4) | |  |
|  | 1 | | 5,118 (42.1) | 709 (51.6) | |  |
|  | 2 | | 1,160 (9.5) | 297 (21.6) | |  |
|  | 3 (most ill) | | 250 (2.1) | 73 (5.3) | |  |
| Any enteral feed in the first 4 days n (%) | | | 11,481 (94.4) | 1,233 (89.7) | |  |
| Transferred units in first 72 hours n (%) | | | 1,812 (14.9) | 296 (21.5) | |  |
| Level of neonatal unit on postnatal day 3  n (%) | | NICU | 9,312 (76.6) | 1,227 (89.2) | |  |
|  |  | Local neonatal unit | 2,787 (22.9) | 134 (9.7) | |  |
|  |  | Special care unit | 53 (0.4) | 9 (0.7) | |  |
| Postnatal day of first enteral feed Median (IQR) | | | 2 (2-3) | 3 (2-4) | |  |
| Days to full feeds Median (IQR) | | | 13 (10-16) | 19 (13-27) | |  |
| Exposed to antibiotics in first 3 postnatal days n (%) | | | 11,311 (93.0) | 1,273 (92.6) | |  |

Proportion of missing data is Caesarean section (5.9%), Maternal ethnicity (22.8%), Antenatal steroids (0.2%), IMD quintile (5.9%), Illness severity score (0.4%), Level of neonatal unit on postnatal day 3 (0.1%), Postnatal day of first enteral feed (1.3%) and Days to full feeds (3.3%).

# REFERENCES

1. Patel N, NeoTrips CG, Evans K, Berrington J, Szatkowski L, Costeloe K, et al. How frequent is routine use of probiotics in UK Neonatal Units? 2023.

2. Cole TJ, Wright CM, Williams AF. Designing the new UK–WHO growth charts to enhance assessment of growth around birth. Archives of disease in childhood Fetal and neonatal edition. 2012;97(3):F219-F22.

3. Appel C. Understanding the Index of Multiple Deprivation (IMD) in public health research 2024 [Available from: <https://www.heec.co.uk/resource/understanding-the-index-of-multiple-deprivation-imd-in-public-health-research/>.

4. Neonatal critical care - service specification: NHS England; 2024 [Available from: <https://www.england.nhs.uk/wp-content/uploads/2015/01/Neonatal-critical-care-service-specification-March-2024.pdf>.
