# Supplementary material for: Description of probiotic use in preterm infants in England and Wales 2016–2022
Source: BMJ Paediatr Open. 2025 Jul 24;9(1):e003605. doi: 10.1136/bmjpo-2025-003605 (PMC12306256; doi:10.1136/bmjpo-2025-003605)
Supplement: Supplementary file 2 [file bmjpo-2025-003605.reviewer_comments.pdf]

PEER REVIEW HISTORY

BMJ Paediatrics Open publishes all reviews undertaken for accepted manuscripts. Reviewers are asked to complete a checklist review form and are provided with free text boxes to elaborate on their assessment. These free text comments are reproduced below.

ARTICLE DETAILS

**TITLE (PROVISIONAL)**	A description of probiotic use in preterm infants in England and Wales 2016-2022
**AUTHORS**	Aveline, Alice; Szatkowski, Lisa; Berrington, Janet; Costeloe, Kate; Bottle, Alex; Ojha, Shalini; Fleming, Paul; Battersby, Cheryl

VERSION 1 - REVIEW

**REVIEWER NAME**	Richard Nicholl
**REVIEWER AFFILIATION**	Northwick Park Hospital, Neonatal Unit
**REVIEWER CONFLICT OF INTEREST**	N/A
**AI DISCLOSURE**	No
**DATE REVIEW RETURNED**	19-May-2025

**GENERAL COMMENTS**	This paper describes the changing use of probiotics in neonatal intensive care units (NICUs), in England and Wales over a 7 year period.<br>Page 3, L2: I would suggest change "neonatal units" to "neonatal intensive care units" as the demographic will be clearer to the reader<br><br>The definition of a "probiotic unit" is rather arbitrary. It is stated that in those units 23% did not receive. There is a limit to how much narrative can be extracted from data.<br>From personal experience it is very likely that individual Consultant behaviour and preference will vary within units (ie the concept of a "probiotic neonatal unit" is a bit<br>fanciful).<br>The PiPs study (and others) showed that cross contamination of treated babies to non treated babies is common. While its not possible to extract from this data, this possibility should be mentioned.<br>Page 4,L49 the Patel survey was of all levels of care, I believe (NICUs, LNUs and special care units). This should be explicitly stated.<br><br>Page 4, L 52 "survey results...may not reflect adherence to guidelines at an infant level" . I am not sure what is meant here. Id suggest a change to something like "adherence by individual care givers" (in reality the decision to use probiotics is at Consultant level.)<br><br>Page 8, table 1: the table is very busy. Is it all necessary? eg is the ethnicity of the mother relevant to the decision to give probiotics?<br><br>Page 12, L236 "limitations": the fact that only NICUs were analysed is a limitation.<br>Page 13,L256: the last line is a bit unfair on probiotic use. Another conclusion might be "probiotic use is widespread and increasing in

	UK NICUs .Our data may help to inform the decision to use in those NICUs currently not doing so".<br><br>see also "Pediatrics" March 2025:<br>Safety and Efficacy of Probiotics for Preterm Infants<br>Roger F. Soll, MD Corresponding Author; Erika M. Edwards, PhD, Roger F. Soll, MD, Vermont Oxford Network, 33 Kilburn St, Burlington, VT 05401. roger.soll@uvmhealth.org, 802-865-4814<br>Pediatrics (2025) 155 (3): e2024069450.<br>https://doi.org/10.1542/peds.2024-069450

**REVIEWER NAME**	Aniko Deierl
**REVIEWER AFFILIATION**	Imperial College Healthcare NHS Trust, Neonatology
**REVIEWER CONFLICT OF INTEREST**	N/A
**AI DISCLOSURE**	No
**DATE REVIEW RETURNED**	21-May-2025

**GENERAL COMMENTS**	This study aimed to describe patterns of probiotic use in preterm infants (<32 weeks gestation) across neonatal units in England and Wales, and to explore factors influencing probiotic exposure. A retrospective cohort analysis was conducted using data from the National Neonatal Research Database (NNRD), covering a 7 year period from 2016 to 2022.<br>Key Findings of the paper<br>• Probiotic Use Trends: The proportion of infants receiving probiotics increased markedly from 9.4% in 2016 to 54.4% in 2022.<br>• Timing of Administration: 90% of probiotic recipients received their first dose within 14 days of birth; 65% by day 5. The median start day was 5, which is notably later than in significant majority of previous RCTs, where administration began earlier once enteral feeding was initiated. Delayed start of probiotic may reduce the positive impact observed in the RCTs soon after birth<br>• Strain Usage: Labinic (LB) was the predominant product until 2020. Use of ProPrems (PP) increased rapidly thereafter, although LB remained the most commonly used strain by the end of the study period.<br>• Variation in Practice: 23% of eligible infants in NICUs that routinely used probiotics did not receive them. This is particularly notable given that probiotics are not contraindicated and considered to be safe when minimal enteral feeding is tolerated.<br>• Factors Influencing Use: Lower gestational age, lower birthweight z-score, and higher illness severity were associated with delayed or absent probiotic administration.<br>• Timing Relative to Feeding: 15.4% of infants received probiotics before their first enteral feed. 66.6% received them after feeding had commenced.<br><br>The authors concluded that probiotic use has increased substantially but remains inconsistent across UK units. Sicker infants are less likely to receive early probiotics, possibly due to clinician hesitancy or perceived instability. Observational data such as this can inform the design of future RCTs and guide policy development.<br>This is a well-conducted and timely study that provides valuable insights into real-world probiotic use in UK based neonatal care. The use of a large, national dataset enhances the robustness of the findings, and the analysis is both comprehensive and clinically relevant.

	Strengths:<br>• Large Cohort: Inclusion of over 48,000 infants enhances generalisability and statistical power.<br>• Real-World Relevance: Reflects actual clinical practice rather than idealised guideline adherence.<br>• Detailed Stratification: Analysis by gestational age, illness severity, and timing of administration provides nuanced insights.<br><br>Limitations<br>•Data Quality: As with all retrospective studies, reliance on routinely collected data may introduce inaccuracies.<br>•Missing Ethnicity Data: While not central to the study's primary question, the high level of missing ethnicity data limits broader equity analyses.<br>• Causality: The observational design precludes definitive conclusions about efficacy or safety, which may concern some clinicians.<br><br>Suggestions<br>• The authors classified recipients as early recipients (within the first 14 days) and late recipients. Further clarification required why the 14 days cut off was chosen, it does not seem to have a scientific basis based on the known evidence. This is significant time from birth and data shows that delayed initiation of probiotics especially in association with high level of antibiotic used during the first week may abolish the positive impact on microbiome and NEC. The authors also comment on this in the discussion in relation to the observed NEC and LOS onset in RCTs. It should be considered to use 7 days of life as classification for early and late recipients for the analysis.<br>• Expand the discussion on the different product use, their compliance with ESPGHAN guidance, the recent FDA alert referenced in the paper. Cost of the product may also have an impact on the choice.<br>• Expand discussion on the implications of delayed administration in sicker infants and potential reasons for infants not exposed to probiotics in units using this treatment. Is there any data suggesting these infants had feed intolerance?<br>• Offer more explanation to better understand barriers to early probiotic use in more premature and sicker infants deviating from evidence form RCTs.<br><br>Recommendation: Accept with minor revisions.<br>This manuscript makes a significant contribution to the neonatal literature and highlights important gaps between policy and practice.

VERSION 1 – AUTHOR RESPONSE

Reviewer comment	Response
**Reviewer 1**	

1. Page 3, L2: I would suggest change "neonatal units" to "neonatal intensive care units" as the demographic will be clearer to the reader	The term “Neonatal intensive care units” in the UK is strictly describing level 3 tertiary centres (level 2=local neonatal units, level 1=special care units), hence we opted for the term neonatal units, as we included all neonatal units in England and Wales in the study.<br><br>The only analysis where we included only NICUs was in the second analysis where we explored factors for withholding probiotics in NICUS that have a probiotic protocol. We had added clarity by<br><br>1) Adding the following paragraph into the introduction to explain the UK neonatal service organisation for international readers:<br><br>“In the UK, neonatal services are organised in geographically defined operational delivery networks (ODNs) and infants are transferred between neonatal units within an ODN to receive appropriate care. Neonatal units are divided into three types: neonatal intensive care units (NICUs) (level 3), local neonatal units (level 2) and special care units (level 1). All infants born before 27 weeks gestation and multiple pregnancies delivered before 28 weeks gestation receive their early days care in a NICU. Probiotic protocols vary within and between networks.”<br><br>2) We have changed the wording to Probiotic Neonatal Intensive Care Units (NICUs) and have also amended the wording to clarify that this applies only for the analysis which explores the factors which influence probiotic exposure other than unit policy on probiotics. Revised wording with changes in red reads:<br><br>Subheading changed to: “Analysis exploring factors influencing probiotic exposure in a ‘probiotic unit’.<br><br>The main determinant of an infant’s probiotic exposure status is whether the unit providing care had implemented a guideline for the routine use of probiotics among preterm infants. To explore other factors that may influence an infant’s exposure to probiotics, we conducted a second analysis excluding units that do not use probiotics routinely. We limited this analysis to “Probiotic NICUs”, defined as a NICU where either the existence of a probiotic guideline was confirmed by that NICU in a survey [10] or data

	analysis confirmed that more than half the infants < 32 weeks admitted in the same month received probiotics. [13]. As NICUs care for larger numbers of very preterm infants each month than local neonatal units or special care units, we could be more confident of correctly classifying NICUs' probiotic status than for units with a smaller monthly denominator. We contacted units where there was uncertainty about their probiotic status from the NNRD data to verify their probiotic status (supplementary materials 3). Infants cared for in a probiotic NICU on postnatal day three were included in this analysis."
2. The definition of a "probiotic unit" is rather arbitrary. It is stated that in those units 23% did not receive. There is a limit to how much narrative can be extracted from data.<br>From personal experience it is very likely that individual Consultant behaviour and preference will vary within units (ie the concept of a "probiotic neonatal unit" is a bit fanciful).	Whilst individual clinicians and families can decide at their discretion whether to administer probiotics to an individual baby, our survey and that by Patel et al show that units either do or do not have a protocol for probiotic use. It is very rare units that do not have a protocol for probiotic use to have suitable probiotics stocked in their pharmacy for neonatal use.<br><br>However, we appreciate that in practice, unit protocols may not be adhered to, which is the reason we conducted the analysis in this study to explore the factors that influenced probiotic exposure in neonatal units that do use probiotics.<br><br>To provide some additional support for this assertion we have included the following (additional wording in red below) in Results subheading "infants cared for in probiotic NICUs" at line 251:<br><br>*"11,262 infants (23.4% of the cohort) were cared for in probiotic NICUs on day three of life and 77.3% (8,701/11,262) of these infants were exposed to probiotics. In contrast, 13.1% (4,835/36,786) of infants cared for in non-probiotic units were exposed to probiotics."*
3. The PiPs study (and others) showed that cross contamination of treated babies to non treated babies is common. While its not possible to extract from this data, this possibility should be mentioned.	We have added the following in the discussion:<br><br>*The PiPs trial [19] demonstrated that cross-contamination between infants who do and do not receive probiotics may occur within units. Therefore we would recommend that future observational studies perform sensitivity analyses that take account of possible cross contamination, by assigning*

	exposure at a unit level, rather than simply at an individual level.
4. Page 4,L49 the Patel survey was of all levels of care, I believe (NICUs, LNUs and special care units). This should be explicitly stated.	We have added the word 'all' to the sentence describing the Patel survey – see below.<br><br>*In 2022, Patel et al [8] conducted a survey of probiotic use in all UK neonatal units which reported that 44% units (n=70/161) routinely used probiotics in the population of preterm infants born at < 32 weeks GA.*<br><br>We have also clarified that our study includes some analyses of all units and some analyses of just NICUs (point 1 above).
5. Page 4, L 52 "survey results...may not reflect adherence to guidelines at an infant level" . I am not sure what is meant here. Id suggest a change to something like "adherence by individual care givers" (in reality the decision to use probiotics is at Consultant level.)	We have amended this language (amendments in red beginning line 62) to address this point:<br><br>Whilst survey results can be helpful to explore differences in practice, they may not reflect adherence by individual caregivers to unit protocols at an infant level.
6. Page 8, table 1: the table is very busy. Is it all necessary? eg is the ethnicity of the mother relevant to the decision to give probiotics?	Given the uncertainty of what influences probiotic exposure in a probiotic NICU, and the potential for individual clinician biases, we believe it is important to retain all the variables including the infant and maternal characteristics. Maternal characteristics like ethnicity have been shown to influence neonatal and maternal mortality and morbidity and could have influenced other care practices, such as probiotic provision.<br><br>However, we have removed the two middle columns from the table to address this point and moved these two columns to a separate supplementary table. We have also removed all the rows describing levels of missing data and substituted the overall percentage of missing data as a footnote in the table. We have consolidated the rows relating to caesarean sections into a single row.

7. Page 12, L236 "limitations": the fact that only NICUs were analysed is a limitation.	The paper is not limited to only NICUs; this was only for the analysis that explored factors that influenced probiotic exposure in Probiotic NICUs.
8. see also "Pediatrics" March 2025: Safety and Efficacy of Probiotics for Preterm Infants<br>Roger F. Soll, MD Corresponding Author; Erika M. Edwards, PhD, Roger F. Soll, MD, Vermont Oxford Network, 33 Kilburn St, Burlington, VT 05401. roger.soll@uvmhealth.org, 802-865-4814<br>Pediatrics (2025) 155 (3): e2024069450. https://doi.org/10.1542/peds.2024-069450	Thank you for this reference. We have included it in the introduction at line 49.
**Reviewer 2**	
1. The authors classified recipients as early recipients (within the first 14 days) and late recipients. Further clarification required why the 14 days cut off was chosen, it does not seem to have a scientific basis based on the known evidence. This is significant time from birth and data shows that delayed initiation of probiotics especially in association with high level of antibiotic used during the first week may abolish the positive impact on microbiome and NEC. The authors also comment on this in the discussion in relation to the observed NEC and LOS onset in RCTs. It should be considered to use 7 days of life as classification for early and late recipients for the analysis.	We appreciate that day 14 may not be viewed as early receipt. We have therefore removed the language about early and late recipients from the paper and simply described the two groups as those receiving probiotics on or before day 14 and those who first receive probiotics after day 14.<br><br>We believe the decision to have a 14 day cut-off is supported by our results; 90% of probiotic recipients receive probiotics in the first 14 days. The group consisting of infants who received probiotics before day 14 therefore represents the routine experience of infants who receive probiotics. If we had restricted our definition to 7 days then 72% of probiotic recipients would have been classified as early recipients and 28% as late recipients.<br><br>We hope that by removing the term early and late, this avoids the misunderstanding that we are suggesting before 14 days is “earlier” than expected or “later” is later than expected.
2. Expand the discussion on the different product use, their compliance with ESPGHAN guidance, the recent FDA alert referenced in the paper. Cost of	We have added this wording in the discussion to address this point:

the product may also have an impact on the choice.	*The choice of probiotic product may be informed by international consensus guidance, national guidelines and other factors such as cost. Our results show that, since the introduction of the probiotic product PP in June 2020, the numbers of infants exposed to this product has been increasing (Figure 3). The current situation, where two products are widely used, provides opportunities for observational studies to explore questions of relative efficacy in the prevention of NEC and probiotic sepsis, a potential risk and central concern of the US FDA.*<br><br>We have also included wording in the introduction to detail the strains of probiotics present in the two most common products.<br><br>*"the two most commonly used probiotics in the UK are a combination of Bifidobacterium infantis, Streptococcus thermophilus and Bifidobacterium lactis (abbreviated as PP) and a product containing Lactobacillus acidophilus, Bifidobacterium bifidum and Bifidobacterium longum spp infantis (abbreviated as LB)."*
3. Expand discussion on the implications of delayed administration in sicker infants and potential reasons for infants not exposed to probiotics in units using this treatment. Is there any data suggesting these infants had feed intolerance?	We have added information about time to full feeds in Table 1 and the following text at line 293 to address this point:<br><br>*The descriptive data shows that median time to first enteral feed is similar in infants who receive their first probiotic after day 14 compared to those who receive probiotics on or before day 14 (day 3 vs day 2). However 25% of the group who first receive probiotics after day 14 have their first enteral feed on or after day 5 and this group also reach full feeds much later than infants who receive probiotics on or before day 14 (day 22 vs day 13). This may suggest feed intolerance contributing to delays in probiotic administration.*
4. Offer more explanation to better understand barriers to early probiotic use in more premature and sicker infants deviating from evidence form RCTs.	Our results reinforce the reviewer's view that there remains equipoise amongst clinicians in neonatal units that have probiotics protocols about the use of probiotics in the most vulnerable preterm infants. We agree future research examining the potential barriers to early probiotic receipt (sicker infants, delayed

	feeding, clinician concern re infection, accidental omission) would be helpful. We believe that observational studies offer an opportunity to address the gaps in the probiotic evidence base and we hope that our forthcoming study examining the efficacy of probiotics in preventing NEC will be a helpful contribution.

VERSION 2 - REVIEW

**REVIEWER NAME**	Richard Nicholl
**REVIEWER AFFILIATION**	Northwick Park Hospital, Neonatal Unit
**REVIEWER CONFLICT OF INTEREST**	N/A
**AI DISCLOSURE**	No
**DATE REVIEW RETURNED**	24-Jun-2025

**GENERAL COMMENTS**	Happy to accept with the changes that have been made.Best wishes.

**REVIEWER NAME**	Aniko Deierl
**REVIEWER AFFILIATION**	Imperial College Healthcare NHS Trust, Neonatology
**REVIEWER CONFLICT OF INTEREST**	N/A
**AI DISCLOSURE**	No
**DATE REVIEW RETURNED**	28-Jun-2025

**GENERAL COMMENTS**	The minor edits in the manuscript helped more accurate understanding of the topic. This paper remains valuable for sharing information about practical aspects of probiotics use in UK NICUs compared to the use in RCT settings. Congratulation to the authors for this work
